# Up-regulation and subcellular localization of hnRNP A2/B1 in the development of hepatocellular carcinoma

**DOI:** 10.1186/1471-2407-10-356

**Published:** 2010-07-06

**Authors:** Huaqing Cui, Feng Wu, Yanling Sun, Guocai Fan, Qingming Wang

**Affiliations:** 1 State Key Laboratory of Proteomics, Beijing Proteome Research Center, Beijing Institute of Radiation Medicine, No. 33 Life Science Park Road, Beijing 102206, China; 2 Department of Hepatology, No. 302 Hospital of PLA, Beijing 100039, China

## Abstract

**Background:**

Hepatocellular carcinoma (HCC) is one of the world's leading causes of death among cancer patients. It is important to find a new biomarker that diagnoses HCC and monitors its treatment. In our previous work, we screened a single-chain antibody (scFv) N14, which could specifically recognize human HepG2 HCC cells but not human non-cancerous liver LO2 cells. However, the antigen it recognized in the cells remained unknown.

**Methods:**

Recombinant scFv N14 antibody was expressed as an active antibody. Using this antibody with a combination of immunological and proteomic approaches, we identified the antigen of scFv N14 antibody as the heterogeneous nuclear ribonucleoprotein A2/B1 (hnRNP A2/B1). The expression of hnRNP A2/B1 in HCC cells was then investigated by semi-quantitative RT-PCR and immunohistochemistry.

**Results:**

We found that the up-regulation of hnRNP A2/B1 was measured at both transcriptional and translational levels in rat HCC cells but not in rat hepatic cells. We also found that in various human hepatic tissues, hnRNP A2/B1 was highly expressed in both human hepatitis virus positive liver tissues and human HCC tissues but not in normal liver tissues. Interestingly, we observed that the localization of hnRNP A2/B1 in HCC cells was altered during the development of HCC. In human hepatitis virus infected tissues hnRNP A2/B1 resides exclusively in the nuclei of hepatocytes. However, when the HCC progressed from a well differentiated to a poorly differentiated stage, hnRNP A2/B1 was increasingly localized in the cytoplasm. In contrast, the HCC tissues with hnRNP A2/B1 highly expressed in the nucleus decreased.

**Conclusions:**

This work is the first to show that hnRNP A2/B1 is the antigen specifically recognized by the scFv N14 antibody in HCC cells. The over-expression of hnRNP A2/B1 was confirmed in cultured human and rat HCC cell lines, human virus related hepatitis liver tissues and human HCC tissues. The increased localization of hnRNP A2/B1 in the cytoplasm of HCC cells was revealed during the dedifferentiation of hepatocellular carcinoma. Therefore, we suggest that the increased expression and cytoplasmic localization of hnRNP A2/B1 can be used as a diagnostic biomarker to assess the risk of human liver cancer.

## Background

Hepatocellular carcinoma (HCC) is one of the world's most common types of cancer, and an estimated 500,000 to 1,000,000 patients die of HCC each year [1]. HCC diagnosis is a multistage process, which include clinical, laboratory, imaging and pathological examinations. Current HCC diagnostic approaches have their limitation. Histopathological examination is considered as the most reliable diagnosis of HCC, but a combination of pathological techniques will certainly improve diagnostic performance [2]. Furthermore, accurate prediction of the invasive potential of HCC is very important for the HCC risk stratification and treatment monitoring [3].

We have been working with screening human HCC cell specific antibodies in order to deliver some efficient biomarkers for the prevention, diagnosis and treatment of HCC. We previously constructed a single-chain antibody library to obtain some hepatoma cell-specific antibodies [4]. We immunized BALB/c mice with HepG2 HCC cells and then isolated total RNA from the spleens. V_H _and V_L _genes were amplified from the total RNA and cloned into phagemids (pCANTAB5E). The recombinant phagemids were transformed to *E. coli *TG1 to construct a mouse phage display library containing 1.1 × 10^6 ^different clones. This library was screened with HepG2 cells, which led to the isolation of a hepatoma cell-specific antibody from a single-chain Fv antibody library termed N14 (scFv N14). However, the specific antigen for this scFv antibody was unknown.

In this study, we report the identification of hnRNP A2/B1 as the antigen recognized by the scFv N14 antibody. A literature search showed that hnRNP A2/B1 is a nuclear RNA-binding protein involved in the splicing of mRNA and its subsequent transport from the nucleus to the cytoplasm [5,6]. hnRNP A2 and hnRNP B1 are produced by alternative splicing of a single-copy gene, and differ from each other only by an additional 12-amino acid insertion at the N-terminus of B1[5,6]. In 1996, Zhou *et al *first reported that hnRNP A2/B1 was the principal antigen for the lung cancer-specific monoclonal antibody 703D4 [7]. Later, hnRNP A2/B1 has been found as the antigen of another antibody MG7 that specific to human gastrointestinal cancers [8]. hnRNP A2/B1 has been reported to be over-expressed in several human cancers, including lung cancer [9,10], colon cancer [11], breast cancer [12], pancreatic cancer [13], and stomach cancer [14].

hnRNP A2/B1 is known as a nuclear RNA-binding protein, but there is an uncertainty of the mis-location of hnRNP A2/B1 in various cells. Different subcellular localizations of hnRNP A2/B1 have been reported in various cases. In cultured cancerous cells, actinomycin D and the methyltransferase inhibitor adenosine dialdehyde can induce nucleocytoplasmic shuttling of hnRNP A2/B1 or hnRNP A2 [15,16]. In human tissues, different subcellular localizations of hnRNP A2/B1 were also observed. Man *et al *reported various subcellular localizations of hnRNP A2/B1 among histologically different cells in the longitudinal section of a small bronchiole [17]. In mammalian lung development, hnRNP A2/B1 was present predominantly in the cytoplasm, but was sometimes also present in the nucleus depending on cell types [18].

Therefore, after we identified hnRNP A2/B1 as the antigen recognized by scFv N14 antibody, we further investigated the expression and subcellular localization of hnRNP A2/B1 in the tumor-derived hepatic cell lines and various human liver tissues samples.

## Methods

### Cell lines and tissue samples

Human HCC cell line HepG2 (ATCC code, HB-8065), QGY-7701 (Cell Bank No. TCHu42), QGY-7703 (Cell Bank No. TCHu43), SMMC-7721 (Cell Bank No. TCHu52), human non-cancerous liver cell line LO2, rat HCC cell lines CBRH-7919 (Cell Bank No. TCR 2) and RH-35 (Cell Bank No. TCR 4) were obtained from the Chinese Academy of Science, Shanghai Cell Library. Specimens from both normal and diseased liver tissues were obtained from the Department of Pathology, No. 302 Hospital, China. The study was performed in accordance with the Helsinki declaration, and informed written consent was obtained from all patients before surgery or liver biopsy. 6 normal human liver samples were both HBsAg and HCVAb negative. In 10 human hepatitis samples, nine were positive for HBsAg with only one was positive for HCVAb. 54 human HCC tissue samples were all positive for HBsAg. The clinical data of the human hepatitis and HCC samples was shown in Table S1 of the additional file 1. All tissue samples were collected, fixed in formalin and embedded in paraffin. Histological differentiation grades for HCC were determined using the Edmondson and Steiner scale [19]. The 54 HCC samples were categorized as well-differentiated (12 cases, Edmondson grade I), moderately-differentiated (27 cases, Edmondson grade I-II and II) or poorly-differentiated (15 cases, Edmondson grade II-III and III). Each sample was reviewed by at least two pathologists specializing in hepatology.

### Isolation rat hepatocytes

Rat hepatocytes were isolated from the livers of female Wistar rats (200~240 g) using collagenase perfusion. After anesthetizing the mice with sodium pentobarbital (100 mg/kg), the liver was first perfused via the portal vein with Ca^2+^-free Krebs-Henseleit (K-H) buffer, then cut into small pieces and digested with collagenase for 30 min at 37°C. The resulting suspension was filtered through 200 mesh sieves, centrifuged at 40 × g for 5 min and washed with PBS buffer. Approximately 2 × 10^8 ^hepatocytes were obtained and used in the following experiments. All procedures using animals were conducted in accordance with protocols approved by the Ethics Committee of the Beijing Institute of Radiation Medicine.

### Expression of scFv N14 antibody in *E. coli*

DNA encoding the full length of scFv N14 antibody (719 bp) was amplified by PCR from the phagemid of scFv N14 using the primers (scFvN14-F: CCGGAATTCATGGCCCAGGTCCAGCTGCAG and scFvN14-R: CCGCTCGAGCCGTTTTATCTCCAAC). The PCR products with EcoRI and XhoI restricted sites introduced in the primers at the 5' and 3' ends were digested and cloned into the expression vector of pET 24a(+) (Novagen). The recombinant scFv N14 antibody containing a his_6_-affinity purification tag was then expressed in *E.coli *BL21 (DE3) cells by induction with 0.25 mM isopropyl β-D-thiogalactoside (IPTG) and incubating for 4 h at 18°C. The cultures were harvested by centrifugation and the cell pellets were stored at -80°C.

### Purification and refolding of recombinant scFv N14 antibody

The cell pellets were resuspended in 15 ml binding buffer (20 mM Tris-HCl pH 8.0, 5 mM EDTA and 5 mM DTT). Cells were sonicated on ice and centrifuged at 6,000 rpm for 10 min at 4°C. The recombinant scFv N14 antibody was expressed in inclusion bodies. Therefore, inclusion bodies in the pellets were first washed three times with washing buffer (20 mM Tris-HCl, pH 8.0, 2 M urea, 5 mM EDTA and 5 mM DTT), then resuspended in 20 ml solubilization buffer (20 mM Tris-HCl, pH 8.0, 8 M urea, 5 mM EDTA and 5 mM DTT), then vortexed until the pellets dissolved. The refolding of the bound protein was performed by adding the inclusion bodies to a buffer containing a low concentration of urea (20 mM Tris-HCl, pH 8.0, 1 M urea, 5 mM EDTA and 5 mM DTT) until the final concentration of urea was 2 M. This soluble refolding fraction was incubated at 4°C for 2 days. The cleared lysate was then applied to a Ni^2+ ^NTA-agarose matrix (Qiagen) column equilibrated with binding buffer (20 mM Tris-HCl pH 8.0, 5 mM EDTA and 5 mM DTT). The column was washed using the binding buffer to remove all of the unbound proteins. Then the bound proteins were eluted with a linear gradient of 0-200 mM imidazole. Fractions containing the scFv N14 antibody were collected, concentrated to 20 mg/ml and stored at -80°C.

### Enzyme-linked immunosorbent assay of recombinant scFv N14 antibody

The enzyme-linked immunosorbent assay (ELISA) was used to assess the activity of the recombinant scFv N14 antibody. HepG2 cells and LO2 cells were grown in 96-well plates and fixed with 4% formaldehyde in PBS buffer for 15 min. Cells were blocked with 5% Casien in PBS buffer, and cells were then incubated with recombinant scFv N14 antibody (5 μg/ml) at RT for 2 h. The secondary antibody used was mouse anti-His_6 _antibody (1:3000, Amersham Bioscience). The cells were then incubated with HRP-conjugated goat anti-mouse IgG (1:3000, Jackson ImmunoResearch) and 3,3',5,5'-tetramethylbenzydine (Sigma) was used as the substrate for HRP. The data was measured at 450 nm with a BioRad microplate reader. PBS buffer rather than the recombinant scFv N14 antibody was used in the negative control for both HepG2 cells and LO2 cells.

### Preparation of nuclear or whole cell protein extracts

Nuclear and cytoplasmic proteins were extracted from HepG2 cells using the NE-PER^® ^nuclear and cytoplasmic extraction kit (PIERCE) according to the protocol provided by the manufacturer. For the whole cell extracts, cells were lysed in RIPA extraction buffer (150 mM sodium chloride, 50 mM Tris-HCl, pH 7.4, 1 mM ethylenediaminetetraacetic acid, 1 mM PMSF, 1% Triton X-100, 1% sodium deoxycholate, 0.1% SDS, 5 mg/ml aprotinin and 5 mg/ml leupeptin) and were then centrifuged. The supernatant was used as the whole cell protein extract.

### SDS-PAGE, 2-D electrophoresis and Q-TOF analysis

The HepG2 nuclear protein extract was analyzed by sodium dodecyl sulfate polyacrylamide gel electrophoresis (SDS-PAGE), two dimensional electrophoresis (2-DE). After electrophoresis the gels were stained with either Coomassie brilliant blue R-250 or electroblotted onto a polyvinylidene difluoride (PVDF) membrane for Western blot analysis.

2-DE and Q-TOF analysis were performed according to the method of Xiao *et al *[20]. For 2-DE analysis typically 100 μl of each sample containing about 100 μg of protein was loaded onto an immobilized non-linear pH gradient (IPG) strip, pH 3-10, 7 cm (Amersham Bioscience). The isoelectric focusing (IEF) was carried out with the IPGphor system (Amersham Bioscience) at room temperature as follows: 6 h at 30 V 6 h at 60 V, 30 min at 500 V, 30 min at 1000 V, 10000 Vh at 5000 V. After IEF, the strips were equilibrated with equilibration buffer (6 M urea, 50 mM Tris-HCl, pH 8.8, 30% glycerol, 2% SDS, 1% DTT and a minimal amount of bromophenol blue) for 15 min. The equilibration buffer was then replaced with a similar equilibration buffer, containing 1% iodoacetamine instead of DTT, for another 15 min. The second dimentional electrophoresis was performed at room temperature on a BioRad system using a 12% acrylamide gel at a constant current of 80 V for 15 min, then at 200 V for 45 min. After electrophoresis, the gels were either stained with Coomassie brilliant blue R-250 or electrotransferred onto a PVDF membrane for the Western blot analysis. Protein spots recognized by scFv N14 antibody were excised manually from the Coomassie blue stained gel. The gel slices were destained with 50% ACN/25 mM NH_4_HCO_3_, reduced with 10 mM DTT at 56°C and alkylated in the dark with 50 mM iodoacetamide at room temperature for 1 h. Then the gel plugs were lyophilized and immersed in 15 μL of 10 ng/μL trypsin solution in 25 mM NH_4_HCO_3_. Digestion was kept at 37°C for 15 h. Tryptic peptide mixtures were first extracted with 100 μL 5% TFA and then with the same volume of 2.5% TFA/50% ACN. The extracted solutions were combined, lyophilized and analyzed by LC-MS. Capillary RP-HPLC of peptide mixture was performed on a Micromass CapLC liquid chromatography system. A fused silica tubing (150 mm × 75 μm i.d.) packed with PepMap C18, 3 μm spherical particles with pore diameter 100 Å (LC Packings, Amsterdam, Netherlands) was used. The flow rate was set at 2.5 μL/min and split into ca. 0.2 μL/min prior to pre-column and analytical column. Mobile phase A consisted of water/ACN (95/5, v/v) with 0.1% FA. Mobile phase B consisted of water/TFA (5/95, v/v) with 0.1% FA. The separation was performed by running a non-linear gradient: 4% B, in 0.1~3.5 min for injection; 4~50% B, in 3.5~63.5 min; 50~100% B, in 63.5~73.5 min. The CapLC is coupled on-line with a Q-TOF Micro mass spectrometer (Micromass, Manchester, UK) for detection and protein identification.

### RT-PCR

Semi-quantitative reverse transcription-polymerse chain reaction (RT-PCR) was used to determine the mRNA transcription of hnRNP A2/B1 in primary rat hepatocytes and rat HCC cell lines. The primers for hnRNP A2/B1 and β-actin amplification were designed according to reference[13] with some modifications. They were F-hnRNPA2B1 5'-TTATGGAGGAGGAAGAGGAG-3' and R-hnRNPA2B1 5'-CTGCATCTGCTCTGGTGTCT-3' for hnRNP A2/B1, which give an about 450 bp RT-PCR product. The primers for hnRNPB1 were F-hnRNPB1: 5'-TTATGGAGGAGGAAGAGGAG-3' and R-hnRNPB1: 5'-CTGCATCTGCTCTGGTGTCT-3', these primers are specific to clone the gene of hnRNP B1 but not hnRNP A2, and will give a 900 bp product. The primers for rat β-actin were R-rat-actin: 5'- CTTCTCTTTAATGTCACGCACG-3' and F-rat-actin: 5'-TAGCCATCCAGGCTGTGTTGTC-3', which give about 230 bp product. The total RNA was extracted respectively from isolated rat healthy hepatocytes, cultured rat RH-35 and CBRH 7919 HCC cells, and used for the synthesis of the first cDNA as described in the literature (Yan-Sanders *et al *[13]). The PCR 50 μl reaction mixture consisted of 0.5 μg cDNA, 0.8 μM each of the primers, 50 μM each of dNTP and 1.5 units of Pyrobest™ DNA polymerase. hnRNP A2/B1, hnRNP B1 and β-actin were amplified separately with the same PCR condition. Thirty cycles were performed as follow: 30 s at 95°C, 45 s at 55°C, and 60 s at 72°C. A final extension was performed at 72°C for 10 min. The PCR products were analyzed by electrophoresis on 1.2% agarose gels and visualized by ethidium bromide staining. Bands were detected using a Gel Doc 2000 and intensities were quantified using Quantity One software (Bio-Rad Laboratories). The hnRNP A2 and/or B1 transcript abundance were expressed relative to the control of β-actin.

### Western blot analysis

Western blot analysis was performed using the following antibodies: scFv N14 antibody (5 μg/ml), mouse anti-His_6 _(1:3000, Amersham Bioscience) and HRP-conjugated goat anti-mouse IgG (1:3000, Jackson ImmunoResearch); or commercial polyclonal goat anti-human hnRNP A2/B1 (1:250; Santa Cruz Biotechnology, recognize both human and rat hnRNP A2/B1) and HRP-conjugated rabbit anti-goat IgG (1:1000, Jackson ImmunoResearch). ß-actin (1:200; Neomarker) was used as the control to normalize the expression levels of hnRNP A2 and/or B1 by Quantity One software (Bio-Rad Laboratories). For 2-D Western blot, after the identification using scFv N14 antibody, we washed the Western blot membrane and re-probed with commercial polyclonal goat anti-human hnRNP A2/B1 to prove that scFv N14 antibody and commercial hnRNP A2/B1 antibody could recognize the same spots.

### Immunofluorescence

HepG2 cells were cultured on glass cover slips, fixed for 10 minutes with 4% formaldehyde in PBS buffer, then permeabilized with 0.5% Triton X-100 in PBS buffer for 15 minutes at room temperature. Immunofluorescence analysis was performed using the following antibodies: scFv N14 antibody (5 μg/ml), mouse anti-His_6 _(1:3000, Amersham Bioscience) and FITC-conjugated goat anti-mouse IgG (1:3000, Sigma). Cell nuclei were stained with DAPI (4, 6-diamidino-2-phenylindole). After immunostaining, the samples were observed using an Olympus 1 × 70 fluorescence microscope.

### Immunohistochemistry

Tissue sections were de-paraffinized and pre-incubated with 0.3% H_2_O_2 _for 15 minutes. Polyclonal goat anti-human hnRNP A2/B1 (1:250; Santa Cruz Biotechnology) was used as the primary antibody and biotin-conjugated rabbit anti-goat IgG as the secondary antibody. HRP-conjugated streptavidin was used as the detection reagent. For the negative control, the primary antibody was replaced by PBS buffer. The sections were stained with diaminobenzidine (DAB) and some samples were also stained with hematoxylin. Three sections from each sample were used for this study. The immunochemical staining result was defined as percentage per 100 HCC cells.

### Evaluation of staining

Analyses were performed by two independent groups of pathologists. The tissue sections were first screened at low power (×10), and the five most representative fields were selected. We counted 100 cells. The staining intensity was semiquantitatively evaluated with a four-tiered system: 0 (negative), 1 (weak), 2 (moderate), and 3 (strong). Weak immunoreactivity was defined as minute granules projecting to the cell. Moderate and strong immunoreactivity were diagnosed when a coarser and more intense staining was seen. If more than 5% of cells had weak, moderate and strong staining, then the section was defined as positive.

### Statistical analysis

Statistical analysis was performed using the SAS 9.0 system (SAS Institute Inc.). The data of the expression levels of hnRNP A2/B1 between normal human liver and human hepatitis samples, normal human liver and human HCC samples were analyzed by the Fisher's exact test. Wilcoxon rank sum test was used to show the correlation between hnRNP A2/B1 distribution and four human liver tissues (hepatitis, well-, moderately- and poorly- differentiation).

## Results and Discussion

### Characterization of recombinant scFv N14 antibody

**Figure 1:**
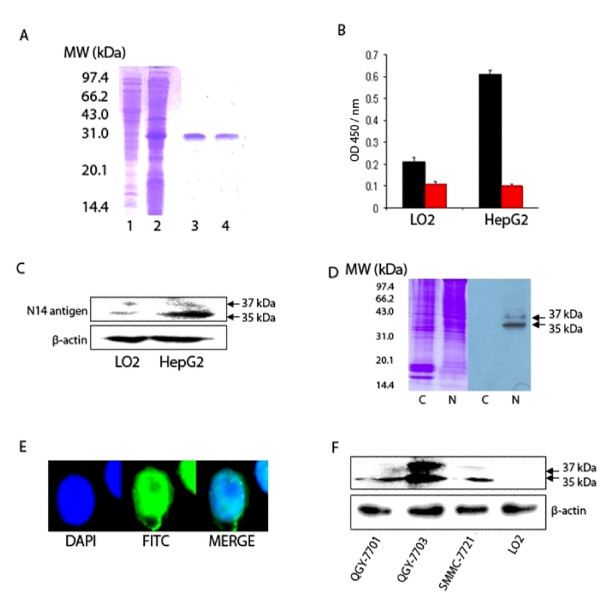
**Characterization of scFv N14 antibody**. **(A) **Purification of recombinant scFv N14 antibody. 1: supernatant of cell lysates of *E.coli *BL21 (DE3); 2: recombinant scFv N14 antibody expressed as inclusion body; 3 and 4, purified refolding scFv N14 antibody. **(B) **ELISA analysis of the binding affinity of scFv N14 antibody to LO2 and HepG2 cells. scFv N14 antibody (black bar) was incubated with LO2 and HepG2 cells, PBS buffer (red bar) was used as the control. **(C) **The expression of scFv N14 antigen in HepG2 cells and LO2 cells. Equal amounts (20 μg) of whole cell extracts from HepG2 cells or LO2 cells were loaded onto the SDS-PAGE gel and normalized by comparing with ß-actin. **(D) **Two protein bands of 35 kDa and 37 kDa in the nuclear protein extract (lane N) of HepG2 cells are recognized by scFv N14 antibody. These bands are not seen in the cytoplasmic protein extract (lane C). **(E) **Immunofluorescence analysis of the sub-cellular localization of scFv N14 antigen within HepG2 cells. Cells were immunostained with scFv N14 antibody, mouse anti-His_6 _antibody and FITC-conjugated goat anti-mouse IgG antibodies. DAPI was used to stain the nuclei. **(F) **The expression of scFv N14 antigen in human HCC cell lines QGY-7701, QGY-7703, SMMC-7721 and human non-cancerous liver LO2 cells. Equal amounts (15 μg) of whole cell protein extracts from various cells were loaded onto the SDS-PAGE gel and normalized by comparing with ß-actin.

The 31 kDa recombinant scFv N14 protein was expressed by the plasmid of pET-24a-scFv N14 in inclusion bodies of *E.coli *BL21 (DE3) (Figure 1A). The renaturation of the recombinant scFv N14 successfully yielded an active recombinant scFv N14 antibody.

The activity of recombinant scFv N14 antibody was measured using ELISA on a typical HCC cell line HepG2 and a normal cell line LO2 as a control (Figure 1B). The results show that the affinity of scFv N14 antibody to HepG2 cells is about three times higher than to LO2 cells. This demonstrated the specificity of the recombinant scFv N14 antibody suitable for the following experiments. Firstly we used this antibody to detect any antigen which could cross-react with scFv N14 antibody by Western blot analysis (Figure 1C). Our data show that recombinant scFv N14 antibody can specifically recognize two bands in the whole cell lysates of both HepG2 cells and LO2 cells. On the gel these two protein bands are much more intense from the HepG2 cells than from LO2 cells. We then further investigated the cellular location of the antigen by cell lysate fraction. Cytoplasmic and nuclear proteins were fractionated from the HepG2 cells, then separated by SDS-PAGE and analyzed by Western blot. The results show that the scFv N14 antibody reacts with two proteins (35 and 37 kDa) in the nuclear fraction but not in the cytoplasmic extract (Figure 1D). This result was further confirmed by immunofluorescent staining the cells that hnRNP A2/B1 was mainly localized in the nuclei of HepG2 cells (Figure 1E).

To investigate whether the scFv N14 antigen is also up-regulated in other HCC cell lines, we chose QGY-7701, QGY-7703 and SMMC-7721 HCC cell lines and the non-cancerous cell line LO2 again as a control, then analyzed the amount of scFv N14 antigen in them by Western blot using scFv N14 antibody. Our data show that the expression of scFv N14 antigen is increased in the three human HCC cell lines but not in LO2 cells and with the highest expression in the QGY-7703 HCC cell line (Figure 1F). Therefore, we conclude that the up-regulation of the scFv N14 antigen is a ubiquitous phenomenon in HCC cells. This leads us to speculate whether the scFv N14 antigen can be used as a new biomarker for human HCC research.

### scFv N14 antibody is specific for hnRNP A2/B1

**Figure 2:**
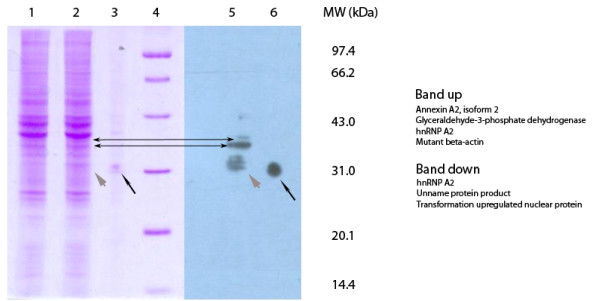
**Identification of scFv N14 antigen**. SDS-PAGE and Western blot analysis of the nuclear protein extract from HepG2 cells showing two bands of 35 kDa and 37 kDa recognized by scFv N14 antibody (indicated by the double-arrows). Lane 1 = nuclear protein extract. Lanes 2 and 5 = nuclear protein extract incorporating a marker protein (His_6_-contain protein, MW = 31.0 kDa, indicated by short gray arrow). Lanes 3 and 6 = marker protein alone (long black arrow). Lane 4 = molecular weight standards. The proteins identified in both two bands (band up and band down) were highlighted on the right.

Our results showed that scFv N14 antigen was enriched in the cell nucleus of HepG2 cells. In order to identity the antigen in HepG2 cell nucleus, we ran the nuclear protein fraction on the SDS-PAGE gel and cut the gel into halves with the lanes of the same loadings, one half of the gel for Western blot and the other half for staining with Coomassie brilliant blue R-250. The Western blot detected two bands with molecular masses of approximately 35 kDa and 37 kDa by scFv N14 antibody (Figure 2). Gel pieces corresponding to the two protein bands were cut out and analyzed by Q-TOF mass spectrometry. Each band contained three or four proteins (Figure 2, for details in the additional files 2 and 3) but only hnRNP A2/B1 was present in both.

**Figure 3:**
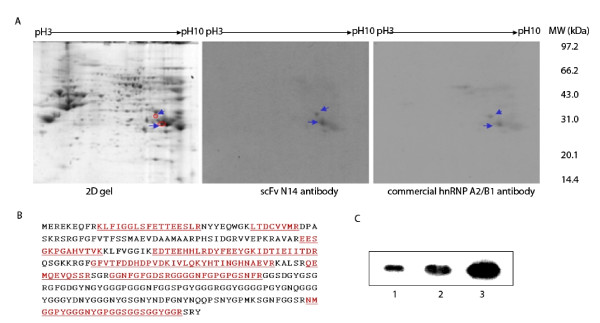
**Identification of scFv N14 antigen by 2D gel/Western blot/MS analysis**. (**A) **Nuclear protein extract of HepG2 cells separated by 2-DE, probed with scFv N14 antibody. Two protein spots reacted with scFv N14 antibody were indicated by arrows in the 2-D gel at molecular weights of 37 kDa (spot up) and 35 kDa (spot down) and pI of around 9.0. After the identification, the Western blot membrane was stripped off the scFv N14 antibody and re-probed with a polyclonal goat anti-hnRNP A2/B1 antibody to prove that scFv N14 and commercial hnRNP A2/B1 could detect the same spots. **(B) **Peptide sequences identified from spot down by Q-TOF analysis. **(C) **Western blot showing scFv N14 bound with recombinant hnRNP A2. Various amounts of hnRNP A2 (Lane 1, 0.4 μg; Lane 2, 0.7 μg; Lane 3, 1.4 μg) were mixed with 20 μg the extracts of *E.coli *Top10 (Invitrogen) and loaded onto the SDS-PAGE gel. ScFv N14 antibody can specifically recognize recombinant hnRNP A2 protein.

We further separated the nuclear proteins using 2-D gel electrophoresis followed by Western blot analysis. Two spots with molecular masses of approximately 37 kDa and 35 kDa with a pI in the range of 8.5-9.5 were identified as hnRNP A2/B1 (Figure 3, for details in the additional files 4 and 5). The Western blot membrane was then stripped and re-probed with a polyclonal goat anti-human hnRNP A2/B1 antibody. The result showed that this antibody bound the same two protein spots as the scFv N14 antibody recognized (Figure 3). Thus the result proves that hnRNP A2/B1 is the antigen for scFv N14 antibody. Interestingly, the antigen for scFv N14 antibody which we identified as hnRNP A2/B1 showed a similar PI and molecular weight to the hnRNP A2/B1 identified by Lee *et al *in cell lysates from the human gastric carcinoma cell line KATO III [14]. We further used our scFv N14 antibody to blot with *E. coli *extract containing the recombinant hnRNP A2 protein [21] and as expected strong binding was observed from the Western blot analysis (Figure 3C).

### hnRNP A2/B1 is up-regulated at both transcriptional and translational levels in proliferative rat HCC cells compared with quiescent rat hepatocytes

**Figure 4:**
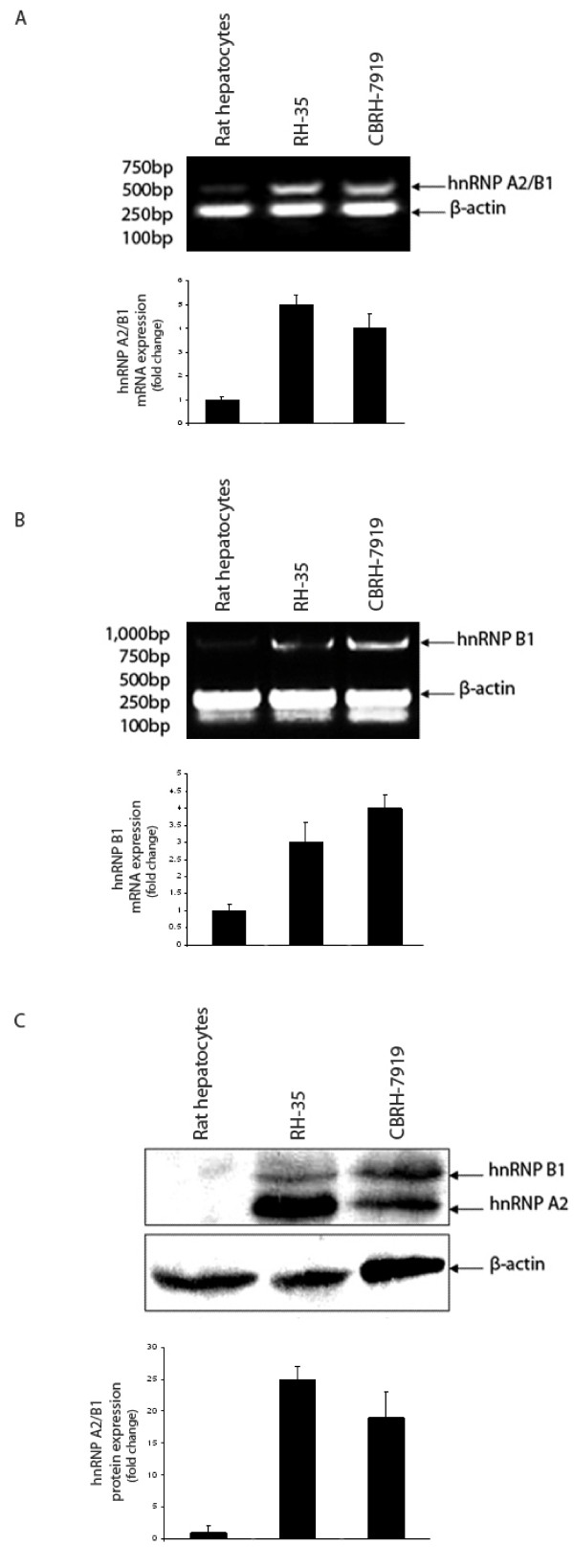
**The over-expression of hnRNP A2/B1 and hnRNP B1 in the mRNA and protein levels in HCC cell lines**. **(A) **The mRNA expression of hnRNP A2/B1 in rat hepatocytes and rat HCC cell lines RH-35 and CBRH-7919. hnRNP A2/B1 mRNA product (450 bp) and β-actin mRNA product (230 bp) are shown in the figure. The blot presents two groups of total 6 independent RT-PCR experiments. The value in the graph is presented as mean ± standard deviation. **(B) **The mRNA expression of hnRNP B1 in rat hepatocytes and rat HCC cell lines RH-35 and CBRH-7919. hnRNP B1 mRNA product (900 bp) and β-actin mRNA product (230 bp) are shown in the figure. The blot presents two groups of total 6 independent RT-PCR experiments. The value in the graph is presented as mean ± standard deviation. **(C) **The protein expression of hnRNP A2/B1 in rat hepatocytes and two rat HCC cell lines. Equal amounts (15 μg) of whole cell protein extracts from rat hepatocytes, rat HCC cell RH-35 and rat HCC cell CBRH-7919 were loaded onto the SDS-PAGE gel and normalized by comparing with ß-actin. Western blot detected two protein bands of 35 kDa (hnRNP A2) and 37 kDa (hnRNP B1) in the whole cell extracts of rat HCC cell lines RH-35 and CBRH-7919. Western blot result is a representative of three independent experiments.

We used semi-quantitative RT-PCR to analyze the transcriptional level of hnRNP A2/B1 and hnRNP B1 at different developmental stages in the isolated healthy rat hepatocytes and rat HCC cell lines. The normal rat hepatocytes were isolated from the healthy liver of the female Wistar rats, which are quiescent cells rather than the proliferative cells. The RT-PCR results show that the mRNA level of hnRNP A2/B1 was up-regulated in two rat HCC cell lines RH-35 and CBRH 7919 compared to rat normal hepatocytes (Figure 4A) and this was also the case for measuring only the mRNA level of hnRNP B1 (Figure 4B), indicating that the mRNA levels of hnRNP A2/B1 or hnRNP B1 are very low in the quiescent stage of rat normal hepatocytes.

The translational levels of hnRNP A2 and hnRNP B1 were analyzed by Western blot respectively (Figure 4C). The results show that hnRNP A2/B1 proteins were up-regulated in two rat HCC cell lines RH-35 and CBRH 7919, but not in rat normal hepatocytes. It was noticed that hnRNP A2 protein was more abundant than hnRNP B1 by 3-5 fold in HepG2, QGY-7701, SMMC-7721 and RH-35 HCC cell lines, but that these two isoforms were equally expressed in HCC cell lines of QGY-7703 and CBRH-7919 (Figure 1F and 4C). Further investigation is required to explain this result.

hnRNP A2 is involved in cell proliferation [22]. Reducing hnRNP A2 expression in Colo16 and HaCaT cells by RNAi led to a non-apoptotic-related decrease in cell proliferation [22]. David *et al *[23] demonstrated that human gliomas overexpressed *c-*Myc, PTB (polypyrimidine tract binding protein), hnRNP A1 and hnRNP A2 to regulate the overexpression of PKM2 (pyruvate kinase isoform 2), which is universally re-expressed in cancer and promotes oxidative aerobic glycolysis [24]. Furthermore, aerobic glycolysis [25] is known to be important for cell growth and tightly regulated in a proliferation-linked manner [26,27]. In this study, we found that hnRNP A2/B1 expression at transcriptional and translational level was up-regulated in the proliferative HCC cells rather than in the quiescent hepatocytes, and this agrees with the fact that the overexpression of hnRNP A2/B1 is required for cell proliferation.

### hnRNP A2/B1 expression is up-regulated in human hepatitis and hepatocellular carcinoma tissue samples

**Figure 5:**
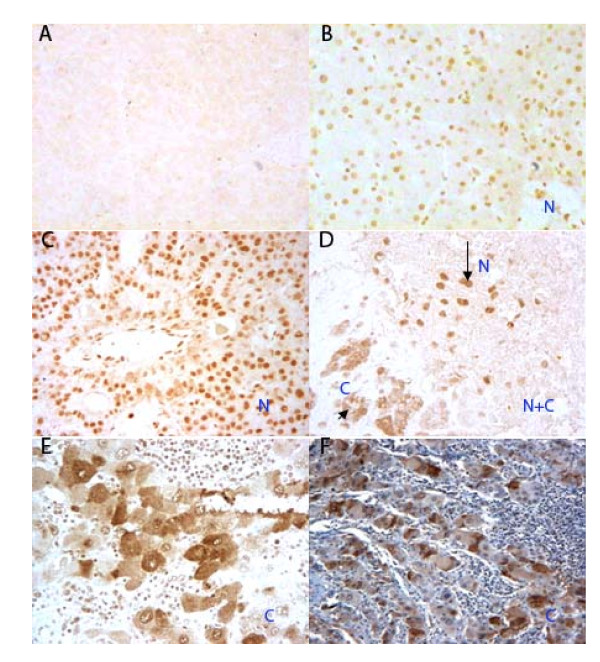
**Immunostaining of hnRNP A2/B1 in human liver tissue samples**. Tissue samples were incubated with a commercial goat polyclonal anti-human hnRNP A2/B1 specific antibody and visualized using diaminobenzidine. **(A)** Human normal liver tissue showing negative staining (×20). **(B)** Hepatitis tissue sample showing positive staining of the hepatocyte nuclei (Marked N) (×20). **(C)** HCC tissue sample showing a nuclear staining pattern (Marked N) (×20). **(D)** HCC tissue sample showing both cytoplasmic (Marked C) (long arrow) and nuclear staining (Marked N) (short arrow) within discrete cell clusters (×20). **(E)** HCC tissue sample showing cytoplasmic staining patterns (Marked C) (×20). **(F)** HCC tissue sample showing a cytoplasmic staining pattern. The cell nuclei have been counterstained with hematoxylin (Marked C) (×10).

An immuno-histochemical approach was used to measure the expression levels of hnRNP A2/B1 in 70 various human live tissues, including healthy liver tissues. The sample information is listed in Table S1 (additional file 1) and the hnRNP A2/B1 expression level is shown in Table 1 and 2. We counted 100 cells in each section and classified the sections into two groups: tissue samples with less than 5% of cells stained were classified as negative; those with 5% or more staining were classified as positive. All of the 6 normal liver tissue samples were negative for hnRNP A2/B1 expression (Figure 5A). In contrast, all 10 hepatitis tissue samples were positive for hnRNP A2/B1 expression (Figure 5B). The 54 HCC tissue samples showed various staining levels for the amount of hnRNP A2/B1 immunoreacted with its specific antibody (Figure 5C-F) and there is none or only marginal staining observed in the peritumoral cirrhotic area of the HCC tissues (data not shown). In all 10 hepatitis tissue samples, we observed the regularity of the granule distribution throughout the whole nucleus without any relation with their pathological stage. However, in the human HCC tissues, the positive immunochemical staining was more intense compared to that of the hepatitis tissues. In general the coarse and thickened granules were mainly dispersed throughout the nucleus, or cytoplasm in cancerous hepatocytes (Figure 5).

**Table 1:** hnRNP A2/B1 expression status in normal human liver versus human hepatitis tissues, and normal human liver versus human HCC tissues by immunohistochemistry

hnRNP A2/B1 expression status	Normal	Hepatitis	HCC
Negative	6	0	5
Positive	0	10	49

Total	6	10	54

1: *P *value of hnRNP A2/B1 expression status between normal human liver samples and human hepatitis samples was <0.01 (Fisher test, statistical analysis see additional file 6). Human hepatitis sample is viral related hepatitis.

2: *P *value of hnRNP A2/B1 expression status between normal human liver samples and human HCC samples was <0.01 (Fisher test, statistical analysis see additional file 6).

5 out of 54 HCC tissue samples showed a very low detectable hnRNP A2/B1 expression and were considered as negative, while the remaining 49 were all positive. Statistical analyses show a significant differences of the expression levels of hnRNP A2/B1 between normal human liver tissues and human hepatitis tissues (Table 1, Fisher test, *P *< 0.01), and between normal human liver tissues and human HCC tissues (Table 1, Fisher test, *P *< 0.01). These immunohistochemistry results show that hnRNP A2/B1 is expressed highly in both hepatitis-positive and HCC liver tissues but not in normal human liver tissues, which is consistent with our results obtained in rat by molecular biochemical approaches (Figure 4).

In our study, we observed that the hnRNP A2/B1 was over-expressed in the cell nuclei of human hepatitis samples. hnRNP A2/B1 was also reported as being over-expressed in both histologically normal and abnormal bronchial epithelial cells from chronic smokers [28]. Hepatitis virus infection and chronic smoking are known factors for the carcinogenesis of human liver cancer and lung cancer respectively [28,29]. In the case of hepatitis virus infection of the liver, continuous inflammation and oxidative stress facilitates the accumulation of genetic alterations within the hepatocytes [29]. hnRNP A2/B1 was indeed found to be involved in the process of DNA repair. Freshly cultured human keratinocytes were irradiated of 100 J/m^2 ^medium wavelength (UV-B), after 6 h, microarray analysis showed that hnRNP B1 mRNA transcript was increased 2.8 fold compared with the control [30]. Whereas, Iwanaga *et al *[31] showed that hnRNP B1 over-expression results in the accumulation of DNA repair errors by inhibiting DNA-dependent protein kinase (DNA-PK) activity. Man *et al *[17] reported that in pulmonary tissue samples hnRNP A2/B1 positive cells contained a significantly higher frequency of microsatellite alteration (MA) and loss of heterozygosity (LOH) compared with cells with no detectable hnRNP A2/B1. Although the mechanisms of hepatocarcinogenesis are still not completely understood, the development and progression of HCC is believed to be the result of accumulated genetic changes. The observation that hnRNP A2/B1 is over-expressed in human hepatitis tissues indicates that hnRNP A2/B1 is involved in the promotion of the hepatocarcinogenesis.

hnRNP A2/B1 has been suggested to be an onco-developmental protein, it was found that within the developing human lung (from 20 weeks onward), hnRNP A2/B1 had the highest expression level in the epithelial cells. However, these levels were reduced in the adult lung [18]. hnRNP A2/B1 is required for cell proliferation [18,24] and contributes to the uncontrolled cell division that is typically seen in cancers [32]. Furthermore, many of its downstream targets are involved in the regulation of the cell cycle and cell proliferation [33]. Other studies showed that small RNA interference targeting of hnRNP A1 and A2 induces cell death in cancer cell lines but not in normal cell lines [34]. Moreover, hnRNP A2/B1 was found to play a role in tumor invasion. Tumorigenic Hep3B cells expressed higher levels of hnRNP A2/B1 than non-tumorigenic HepG2 cells [35]. hnRNP A2 is important in generating appropriate of the Golgi complex, which is required for polarized cell migration and for tumor cell invasion [36,37]. The study of Guha *et al *also suggests that hnRNP A2 is very important in the induction of cell growth and invasiveness stimulated by mitochondrial stress [38,39]. Taking together with our results, we suggest that hnRNP A2/B1 is also required for the proliferation and tumor invasion of HCC.

### Cytoplasmic localization of hnRNP A2/B1 is an indicator of the dedifferentiation of hepatocellular carcinoma

hnRNP A2/B1 is various subcellularly localized in human hepatitis and HCC tissues. We defined 3 patterns of hnRNP A2/B1 subcellular localization. The sample sections with all the cell clusters of nuclear staining were defined as nuclear localization (N) (Figure 5B and 5C); the sections with all the cell clusters of cytoplasmic staining were defined as cytoplasmic localization (C) (Figure 5E and 5F); the sections with both nuclear and cytoplasmic staining observed simultaneously in discrete clusters of cancerous cells within the same sample were defined as both nuclear and cytoplasmic localization (N+C), they contain at least one cluster of cells of nuclear or cytoplasmic staining (Figure 5D). In 10 positive hnRNP A2/B1 staining hepatitis tissue samples (Table 2), hnRNP A2/B1 was exclusively expressed in the cell nuclei (Figure 5B). Whereas, in 49 HCC positive staining tissue samples all three patterns of hnRNP A2/B1 subcellular localization were observed.

**Table 2:** hnRNP A2/B1 subcellular localization in HCC tissues with different histopathological grades by immunohistochemistry

hnRNP A2/B1 distribution	Hepatitis	HCC		
		
		Well	Moderately	Poorly
N	10 (100%)	5 (42%)	5 (22%)	2 (14%)
N + C	0	6 (50%)	9 (39%)	2 (14%)
C	0	1 (8%)	9 (39%)	10 (72%)

Total	10	12	23	14

1: Subcellular localization is described as N, nuclear localization; N+C, both nuclear localization and cytoplasmic localization observed in the same sample; C, cytoplasmic localization;

2: Three groups of HCC tissues were defined as Well-differentiation, Moderately-differentiation, Poorly-differentiation according to the degree of tumor differentiation;

3: *P *value of the correlation between hnRNP A2/B1 subcellular localization and the degree of HCC tumor differentiation, *P *= 0.0001, *P *< 0.05 (Wilcoxon rank sum test, statistical analysis see additional file 6).

**Figure 6:**
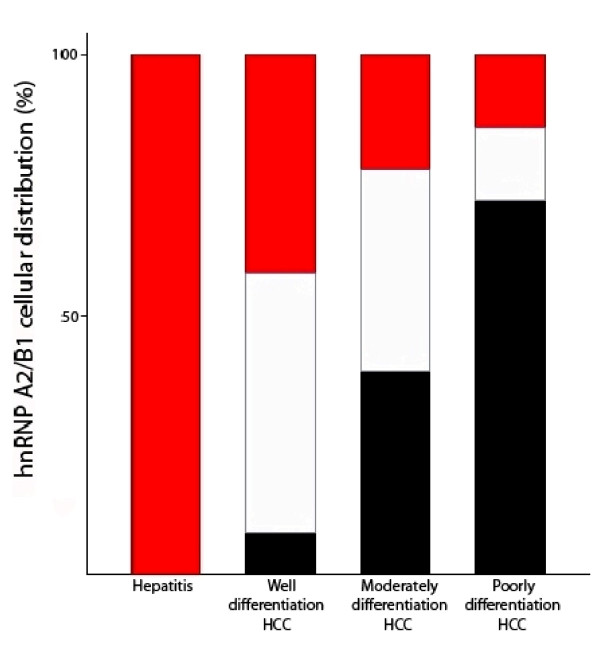
**The subcellular localization of hnRNP A2/B1 in human hepatitis and HCC tissues**. The number of samples defined as nuclear localization (red bar), both nuclear and cytoplasmic localization (white bar) and cytoplasmic localization (black bar) in each group of human liver tissues (viral hepatitis, well differentiation HCC, moderately differentiation HCC and poorly differentiation HCC) was expressed as percentage respectively (Data obtained from Table 2).

According to the developmental stages, 49 immunochemical staining positive human HCC samples were classified into 3 groups: 12 well-differentiated HCC samples, 23 moderately-differentiated and 14 poorly-differentiated (Figure 6 and Table 2). In 12 well-differentiated HCC tissue samples, 8% of them showed hnRNP A2/B1 cytoplasmic localization, 42% nuclear localization and 50% showed both cytoplasmic and nuclear localiztion within discrete cell clusters in the same tissue sample. In 23 moderately-differentiated samples, the percentage of cytoplasmic localized samples increased to 39% while the percentage of nuclear localization, both nuclear and cytoplasmic localization samples decreased to 22% and 39% respectively. Interestingly, in 14 poorly-differentiated HCC samples, 72% of them had cells with hnRNP A2/B1 localized in cytoplasm and 14% in nuclear and the same percentage in both cytoplasmic and nuclear localization (Table 2). Therefore, the above results show a clear increasing trend in the percentage of hnRNP A2/B1 cytoplasmic localization tissue samples from well-differentiated to poorly-differentiated stages (Figure 6).

The results of Wilcoxon rank sum test show a significant correlation (*P *= 0.0001, *P *< 0.05) between the subcellular localization of hnRNP A2/B1 and the different stages of human liver tissues (Table 2). These results suggested that the cell localization of hnRNP A2/B1 from the nucleus to the cytoplasm in the hepatocytes is correlated to HCC development.

During the dedifferentiation of the tumor cells, the malignant potential increases, as reflected in intrahepatic metastasis and vascular invasion. This transformation occurs non-uniformly within a given tumor nodule, resulting in the coexistence of well-differentiated and moderately- to poorly- differentiated lesions within the same nodule. This has been termed by histologists a 'nodule-in-nodule' or 'mosaic' appearance [40]. This might explain the diversity of the hnRNP A2/B1 subcellular localization in human HCC tissues observed in this study.

Nuclear localization of hnRNP A2/B1 in cultured cell lines is known. However, the stimulation by actinomycin D or adenosine dialdehyde will cause the nuclei-cytoplasm translocation of hnRNP A2/B1 [16,41]. Several studies described some possible mechanisms involved in the up-regulation and subcellular translocation of hnRNP A2/B1. Kit Wan *et al *[42] reported that over-expression of EGFP-SUMO-1 (sumoylation-1) will increase the expression of hnRNP B1 in HepG2 cells. Pioli *et al *[43] presented that hnRNP A2 interacts with an ubiquitin-protein isopeptide ligase, pVHL. The post-translational modification of hnRNP A2/B1 might protect the proteins from degradation resulting in the observed high protein expression and subcelluar translocation. Nichols *et al *showed that the translocation of hnRNP A2 to cytoplasm was linked to the pattern of methylation within the RGG domain (R191-G253) by inhibiting the methyltransferase enzyme [16,44], while the research of Bosser *et al *also suggested that the phosphorylation might be another mechanism affects the cellular localization of hnRNP A2 [45].

Man *et al *speculated that subcellular localization of hnRNP A2/B1 might be an important factor associated with tumor progression. They reported that in lung cancer tissues, cells with hnRNP A2/B1 presented in the cytoplasm had a 3-fold higher frequency of MA and LOH than cells with hnRNP A2/B1 confined to the nucleus [17]. Nichols *et al *assumed that the cytoplasmic over-expression of hnRNP A2 in airway epithelial cells was associated with neoplastic transformation and/or tumorigenesis [16]. Interestingly, the various subcellular localizations of hnRNP A2/B1 in human cancer tissues were observed in many cases, however, the isoform hnRNP B1 is up to now reported exclusively to be localized in the nucleus [32,46-49]. Therefore, we speculate that in the poorly-differentiated HCC tissues only the isoform of hnRNP A2 is very likely over-expressed in the cell cytoplasm. hnRNP A2 and hnRNP B1 are two closely related splice variants of the hnRNP A/B family [5], hnRNP A2/B1 are often functionally studied together. There are reported antibodies that recognize hnRNP A2/B1 [10] or hnRNP B1 respectively [41]. In this study, our scFv N14 antibody and commercial hnRNP A2/B1 antibody both exhibited relative limitation considering that they can not distinguish hnRNP B1 from hnRNP A2/B1. It is worthwhile in the future to distinguish the subcellular localization of these two isoforms by using their specific antibodies in immunohistochemical experiments.

## Conclusions

hnRNP A2/B1 was identified as the antigen of the scFv N14 antibody, which specifically recognizes HepG2 HCC cells but not human non-cancerous liver LO2 cells. hnRNP A2/B1 was observed highly expressed at both transcriptional and translational levels in cultured rat HCC cell lines but not in rat hepatocytes. hnRNP A2/B1 has low expression in human normal tissues, but is over-expressed in human hepatitis and HCC tissues. The high expression of hnRNP A2/B1 might promote the hepatocarcinogenesis in these hepatitis patients, and the increased expression of hnRNP A2/B1 is assumed to be required for cell proliferation and tumor invasion. Another interesting phenomena revealed in this study is that, in human hepatitis tissues, hnRNP A2/B1 is exclusively distributed in the nucleus; in most of the well differentiated human HCC tissues, hnRNP A2/B1 mainly accumulates in the nucleus; while in most of the poorly differentiated human HCC tissues, it is predominantly localized in the cytoplasm. The increased cytoplasmic localization of hnRNP A2/B1 is correlated to the progression in de-differentiation of hepatocytes. Considering the complexity of human HCC, we believe that the detection of cytoplasmic over-expression of hnRNP A2/B1 is a very promising diagnostic biomarker to use for HCC risk stratification and treatment monitoring.

## Abbreviations

Heterogeneous nuclear ribonucleoprotein (hnRNP) A2/B1, hepatocellular carcinoma (HCC), single chain Fv antibody (scFv)

## Competing interests

The authors declare that they have no competing interests.

## Authors' contributions

HC carried out the experimental work, analyzed the data and wrote the manuscript. FW help in HC experimental work and performed statistical analysis. YS contributed to analysis of the data of immunohistochemistry. GF help in cell culture. QW designed the experiment, analyzed the data and compiled the manuscript. All authors read and approved the final manuscript.

## Pre-publication history

The pre-publication history for this paper can be accessed here:

http://www.biomedcentral.com/1471-2407/10/356/prepub

## Supplementary Material

Additional file 1 **Table S1: The clinical data of the human tissues (normal, hepatitis and HCC samples)**. The clinical information for the human tissues used in this study. Click here for file

Additional file 2 **The result of Q-TOF mass spectrometry analysis of the band up in Figure **2. Peptide sequences identified from band up by Q-TOF analysis. Click here for file

Additional file 3 **The result of Q-TOF mass spectrometry analysis of the band down in Figure **2. Peptide sequences identified from band down by Q-TOF analysis. Click here for file

Additional file 4 **The result of Q-TOF mass spectrometry analysis of the spot up in Figure **3. Peptide sequences identified from spot up by Q-TOF analysis. Click here for file

Additional file 5 **The result of Q-TOF mass spectrometry analysis of the spot down in Figure **3. Peptide sequences identified from spot down by Q-TOF analysis. Click here for file

Additional file 6 **The statistical analysis results of Table **1**and Table **2. The SAS statistical analysis results. Click here for file

## References

[B1] Bosch FX Ribes J Diaz M Cleries R Primary liver cancer: Worldwide incidence and trends Gastroenterology 2004 127 S5 S16 10.1053/j.gastro.2004.09.011 15508102

[B2] Gomaa AI Khan SA Leen ELS Waked I Taylor-Robinson SD Diagnosis of hepatocellular carcinoma World J Gastroentero 2009 15 1301 1314 10.3748/wjg.15.1301 PMC2658831 19294759

[B3] Kaseb AO Hanbali A Cotant M Hassan MM Wollner I Philip PA Vascular Endothelial Growth Factor in the Management of Hepatocellular Carcinoma A Review of Literature Cancer 2009 115 4895 4906 10.1002/cncr.24537 19637355

[B4] Nian H An L Zhao Y Chen J Fan G Wang Q Construction and screening of Anti-Hepatoma Cells phage display scFv library Current Immunolugy (Chinese) 2004 24 357 361

[B5] Kozu T Henrich B Schafer KP Structure and Expression of the Gene (Hnrpa2b1) Encoding the Human Hnrnp Protein A2/B1 Genomics 1995 25 365 371 10.1016/0888-7543(95)80035-K 7789969

[B6] Carpenter B MacKay C Alnabulsi A MacKay M Telfer C Melvin WT Murray GI The roles of heterogeneous nuclear ribonucleoproteins in tumour development and progression BBA-Rev Cancer 2006 1765 85 100 10.1016/j.bbcan.2005.10.002 16378690

[B7] Zhou J Mulshine JL Unsworth EJ Scott FR Avis IM Vos MD Treston AM Purification and characterization of a protein that permits early detection of lung cancer - Identification of heterogeneous nuclear ribonucleoprotein-A2/B1 as the antigen for monoclonal antibody 703D4 J Bio Chem 1996 271 10760 10766 10.1074/jbc.271.18.10760 8631886

[B8] Gao JN Gao Y Ju YF Yang JJ Wu Q Zhang JC Du XM Wang ZQ Song YB Li H Proteomics-based generation and characterization of monoclonal antibodies against human liver mitochondrial proteins Proteomics 2006 6 427 437 10.1002/pmic.200500409 16342244

[B9] Fielding P Turnbull L Prime W Walshaw M Field JK Heterogeneous nuclear ribonucleoprotein A2/B1 up-regulation in bronchial lavage specimens: A clinical marker of early lung cancer detection Clin Cancer Res 1999 5 4048 4052 10632338

[B10] Wang X Peng XD Li G Hu LJ Bi JH Cloning, ligation and expression of the variable region genes of the monoclonal antibody against human HnRNPA2/B1 Zhonghua Yi Xue Yi Chuan Xue Za Zhi 2004 21 548 551 15583979

[B11] Ushigome M Ubagai T Fukuda H Tsuchiya N Sugimura T Takatsuka J Nakagama H Up-regulation of hnRNP A1 gene in sporadic human colorectal cancers Int J Oncol 2005 26 635 640 15703818

[B12] Zhou J Allred DC Avis I Martinez A Vos MD Smith L Treston AM Mulshine JL Differential expression of the early lung cancer detection marker, heterogeneous nuclear ribonucleoprotein-A2/B1 (hnRNP-A2/B1) in normal breast and neoplastic breast cancer Breast Cancer Res Tr 2001 66 217 224 10.1023/A:1010631915831 11510693

[B13] Yan-Sanders Y Hammons GJ Lyn-Cook BD Increased expression of heterogeneous nuclear ribonucleoprotein A2/B1 (hnRNP) in pancreatic tissue from smokers and pancreatic tumor cells Cancer Lett 2002 183 215 220 10.1016/S0304-3835(02)00168-4 12065097

[B14] Lee CH Lum JHK Cheung BPY Wong MS Butt YKC Tam MF Chan WY Chow C Hui PK Kwok FSL Identification of the heterogeneous nuclear ribonucleoprotein A2/B1 as the antigen for the gastrointestinal cancer specific monoclonal antibody MG7 Proteomics 2005 5 1160 1166 10.1002/pmic.200401159 15759317

[B15] Kamma H Horiguchi H Wan LL Matsui M Fujiwara M Fujimoto M Yazawa T Dreyfuss G Molecular characterization of the hnRNP A2/B1 proteins: Tissue-specific expression and novel isoforms Exp Cell Res 1999 246 399 411 10.1006/excr.1998.4323 9925756

[B16] Nichols RC Wang XW Tang J Hamilton BJ High FA Herschman HR Rigby WFC The RGG domain in hnRNP A2 affects subcellular localization Exp Cell Res 2000 256 522 532 10.1006/excr.2000.4827 10772824

[B17] Man YG Martinez A Avis IM Hong SH Cuttitta F Venzon DJ Mulshine JL Phenotypically different cells with heterogeneous nuclear ribonucleoprotein A2/B1 overexpression show similar genetic alterations Am J Res Cell Mol 2000 23 636 645 10.1165/ajrcmb.23.5.4177 11062142

[B18] Montuenga LM Zhou J Avis I Vos M Martinez A Cuttitta F Treston AM Sunday M Mulshine JL Expression of heterogeneous nuclear ribonucleoprotein A2/B1 changes with critical stages of mammalian lung development Am J Res Cell Mol 1998 19 554 562 10.1165/ajrcmb.19.4.3185 9761751

[B19] Edmondson H Steiner P Primary Carcinoma of the liver: A study of 100 cases among 48,900 necropsies Cancer 1954 7 462 503 10.1002/1097-0142(195405)7:3<462::AID-CNCR2820070308>3.0.CO;2-E 13160935

[B20] Xiao T Ying WT Li L Hu Z Ma Y Jiao LY Ma JF Cai Y Lin DM Guo SP An approach to studying lung cancer-related proteins in human blood Mol Cell Proteomics 2005 4 1480 1486 10.1074/mcp.M500055-MCP200 15970581

[B21] Ling Y Mengxue Y Hongen Y Jiayou L Yang G Xiaxiu H The clone, expression, purification and clinical application of hnRNP A2/B1 Chinese Journal of Microbiology and Immunology 2002 22 4

[B22] He YW Brown MA Rothnagel JA Saunders NA Smith R Roles of heterogeneous nuclear ribonucleoproteins A and B in cell proliferation J Cell Sci 2005 118 3173 3183 10.1242/jcs.02448 16014382

[B23] David CJ Chen M Assanah M Canoll P Manley JL HnRNP proteins controlled by c-Myc deregulate pyruvate kinase mRNA splicing in cancer Nature 2010 463 364 U114 10.1038/nature08697 20010808 PMC2950088

[B24] Christofk HR Vander Heiden MG Harris MH Ramanathan A Gerszten RE Wei R Fleming MD Schreiber SL Cantley LC The M2 splice isoform of pyruvate kinase is important for cancer metabolism and tumour growth Nature 2008 452 230 U274 10.1038/nature06734 18337823

[B25] Warburg O On the origin of cancer cells Science 1956 123 309 314 10.1126/science.123.3191.309 13298683

[B26] Heiden MGV Cantley LC Thompson CB Understanding the Warburg Effect: The Metabolic Requirements of Cell Proliferation Science 2009 324 1029 1033 19460998 10.1126/science.1160809 PMC2849637

[B27] Wang T Marquardt C Foker J Aerobic Glycolysis during Lymphocyte-Proliferation Nature 1976 261 702 705 10.1038/261702a0 934318

[B28] Zhou J Mulshine JL Ro JY Avis I Yu R Lee JJ Morice R Lippman SM Lee JS Expression of heterogeneous nuclear ribonucleoprotein A2/B1 in bronchial epithelium of chronic smokers Clin Cancer Res 1998 4 1631 1640 9676837

[B29] Fung J Lai CL Yuen MF Hepatitis B and C virus-related carcinogenesis Clin Microbiol Infect 2009 15 964 970 10.1111/j.1469-0691.2009.03035.x 19874379

[B30] Takao J Ariizumi K Dougherty II Cruz PD Genomic scale analysis of the human keratinocyte response to broad-band ultraviolet-B irradiation Photodermatol Photo 2002 18 5 13 10.1034/j.1600-0781.2002.180102.x 11982916

[B31] Iwanaga K Sueoka N Sato A Hayashi S Sueoka E Heterogeneous nuclear ribonucleoprotein B1 protein impairs DNA repair mediated through the inhibition of DNA-dependent protein kinase activity Biochem Bioph Res Co 2005 333 888 895 10.1016/j.bbrc.2005.05.180 15964549

[B32] Wu SL Sato M Endo C Sakurada A Dong BM Aikawa H Chen Y Okada Y Matsumura Y Sueoka E Kondo T hnRNP B1 protein may be a possible prognostic factor in squamous cell carcinoma of the lung Lung Cancer 2003 41 179 186 10.1016/S0169-5002(03)00226-5 12871781

[B33] He Yaowu JAR Epis Michael R Leedman Peter J Smith Rose Downstream targets of heterogeneous nuclear ribonucleoprotein A2 mediate cell proliferation Mol Carcinogen 2008 48 167 179 10.1002/mc.20467 18680105

[B34] He Y Brown MA Rothnagel JA Saunders NA Smith R Roles of heterogeneous nuclear ribonucleoproteins A/B in cell growth Mol Biol Cell 2004 15 252A 253A 10.1242/jcs.02448 16014382

[B35] Lee CL Hsiao HH Lin CW Wu SP Huang SY Wu CY Wang AHJ Khoo KH Strategic shotgun proteomics approach for efficient construction of an expression map of targeted protein families in hepatoma cell lines Proteomics 2003 3 2472 2486 10.1002/pmic.200300586 14673797

[B36] Kupfer A Louvard D Singer SJ Polarization of the Golgi-Apparatus and the Microtubule-Organizing Center in Cultured Fibroblasts at the Edge of an Experimental Wound P Natl A Sci USA-Biol 1982 79 2603 2607 10.1073/pnas.79.8.2603 PMC346248 7045867

[B37] Moran-Jones K Grindlay J Jones M Smith R Norman JC hnRNP A2 Regulates Alternative mRNA Splicing of TP53INP2 to Control Invasive Cell Migration Cancer Res 2009 69 9219 9227 10.1158/0008-5472.CAN-09-1852 19934309 PMC6485436

[B38] Amuthan G Biswas G Zhang SY Klein-Szanto A Vijayasarathy C Avadhani NG Mitochondria-to-nucleus stress signaling induces phenotypic changes, tumor progression and cell invasion Embo J 2001 20 1910 1920 11296224 10.1093/emboj/20.8.1910 PMC125420

[B39] Guha M Pan H Fang JK Avadhani NG Heterogeneous Nuclear Ribonucleoprotein A2 Is a Common Transcriptional Coactivator in the Nuclear Transcription Response to Mitochondrial Respiratory Stress Mol Biol Cell 2009 20 4107 4119 19641020 10.1091/mbc.E09-04-0296 PMC2743628

[B40] Kojiro M Nakashima O Histopathologic evaluation of hepatocellular carcinoma with special reference to small early stage tumors Semin Liver Dis 1999 19 287 296 10.1055/s-2007-1007118 10518308

[B41] Kamma H Satoh H Matusi M Wu WW Fujiwara M Horiguchi H Characterization of hnRNP A2 and B1 using monoclonal antibodies: intracellular distribution and metabolism through cell cycle Immunol Lett 2001 76 49 54 10.1016/S0165-2478(00)00318-7 11222913

[B42] Ma KW Au SWN Waye MMY Over-expression of SUMO-1 induces the up-regulation of heterogeneous nuclear ribonucleoprotein A2/B1 isoform B1 (hnRNP A2/B1 isoform B1) and uracil DNA glycosylase (UDG) in hepG2 cells Cell Biochem Funct 2009 27 228 237 10.1002/cbf.1562 19384898

[B43] Pioli PA Rigby WFC The von Hhippel-Lindau protein interacts with heteronuclear ribonucleoprotein A2 and regulates its expression J Bio Chem 2001 276 40346 40352 10.1074/jbc.M105391200 11517223

[B44] Diamandis EP Fritsche HA Lilja H Chan DW Schwart MK Tumor Markers: Physiology, Pathobiology, Technology and Clinical Application 2002 Washington DC: AACC press

[B45] Bosser R Faura M Serratosa J Renaupiqueras J Pruschy M Bachs O Phosphorylation of Rat-Liver Heterogeneous Nuclear Ribonucleoprotein-A2 and Ribonucleoprotein-C Can Be Modulated by Calmodulin Mol Cell Biol 1995 15 661 670 7823935 10.1128/mcb.15.2.661 PMC231926

[B46] Hiraki A Murakami T Aoe K Sueoka E Sueoka N Taguchi K Kamei T Sugi K Ueoka H Kishimoto T Heterogeneous nuclear ribonucleoprotein B1 expression in malignant mesothelioma Cancer Sci 2006 97 1175 1181 10.1111/j.1349-7006.2006.00311.x 16939492 PMC11158878

[B47] Sueoka E Sueoka N Goto Y Matsuyama S Nishimura H Sato M Fujimura S Chiba H Fujiki H Heterogeneous nuclear ribonucleoprotein B1 as early cancer biomarker for occult cancer of human lungs and bronchial dysplasia Cancer Res 2001 61 1896 1902 11280744

[B48] Tani H Ohshima K Haraoka S Hamasaki M Kamma H Ikeda S Kikuchi M Reduced expression of heterogeneous nuclear ribonucleoprotein B1 in adult T-cell lymphoma/leukemia Int J Oncol 2003 22 529 534 12579305

[B49] Sueoka E Goto Y Sueoka N Kai Y Kozu T Fujiki H Heterogeneous nuclear ribonucleoprotein B1 as a new marker of early detection for human lung cancers Cancer Res 1999 59 1404 1407 10197602

