# Supplementary material for: Up-regulation and subcellular localization of hnRNP A2/B1 in the development of hepatocellular carcinoma
Source: BMC Cancer. 2010 Jul 6;10:356. doi: 10.1186/1471-2407-10-356 (PMC2915982; doi:10.1186/1471-2407-10-356)
Supplement: Additional file 1 — Table S1: The clinical data of the human tissues (normal, hepatitis and HCC samples). The clinical information for the human tissues used in this study. [file 1471-2407-10-356-S1.PDF]

Table S1: Clinical data of the healthy (1-6), hepatitis positive (7-16) and HCC (17-70) samples

**Num**	**Gender**	**Age**	**Hepatitis Infection**	**stage**	**Histopathological grade**
Healthy					
1	F	37	NO	-	-
2	M	43	NO	-	-
3	M	32	NO	-	-
4	M	45	NO	-	-
5	M	36	NO	-	-
6	M	52	NO	-	-
Hepatitis					
7	F	43	HBV	-	-
8	M	26	HBV	-	-
9	M	39	HBV	-	-
10	M	11	HCV	-	-
11	M	38	HBV	-	-
12	M	40	HBV	-	-
13	F	42	HBV	-	-
14	M	54	HBV	-	-
15	F	33	HBV	-	-
16	M	15	HBV	-	-
HCC					
17	M	40	HBV	I	well
18	F	45	HBV	I	well
19	M	42	HBV	I	well
20	F	32	HBV	I	well
21	M	52	HBV	I	well
22	M	59	HBV	I	well
23	M	61	HBV	I	well
24	M	49	HBV	I	well
25	M	44	HBV	I	well
26	M	69	HBV	I	well
27	M	33	HBV	I	well

28	M	52	HBV	I	well
29	F	57	HBV	I – II	moderately
30	M	63	HBV	I – II	moderately
31	M	49	HBV	I – II	moderately
32	M	47	HBV	I – II	moderately
33	M	37	HBV	I – II	moderately
34	M	43	HBV	I – II	moderately
35	M	55	HBV	I – II	moderately
36	M	50	HBV	I – II	moderately
37	M	54	HBV	II	moderately
38	M	63	HBV	II	moderately
39	M	48	HBV	II	moderately
40	M	56	HBV	II	moderately
41	M	49	HBV	II	moderately
42	M	43	HBV	II	moderately
43	M	55	HBV	II	moderately
44	F	58	HBV	II	moderately
45	M	56	HBV	II	moderately
46	M	64	HBV	II	moderately
47	M	57	HBV	II	moderately
48	M	39	HBV	II	moderately
49	F	98	HBV	II	moderately
50	F	70	HBV	II	moderately
51	M	40	HBV	II	moderately
52	F	66	HBV	II	moderately
53	M	65	HBV	II	moderately
54	F	57	HBV	II	moderately
55	M	59	HBV	II	moderately
56	M	53	HBV	II – III	poorly
57	M	45	HBV	II – III	poorly
58	M	47	HBV	II – III	poorly
59	M	63	HBV	II – III	poorly
60	M	47	HBV	II – III	poorly

61	F	50	HBV	II – III	poorly
62	M	51	HBV	II – III	poorly
63	M	72	HBV	II – III	poorly
64	F	75	HBV	III	poorly
65	M	49	HBV	III	poorly
66	F	57	HBV	III	poorly
67	F	54	HBV	III	poorly
68	M	48	HBV	III	poorly
69	M	49	HBV	III	poorly
70	F	57	HBV	III	poorly

Degree of tumor differentiation: well: well-differentiation; moderately: moderately-differentiation; poorly: poorly-differentiation.
