# Supplementary material for: Up-regulation and subcellular localization of hnRNP A2/B1 in the development of hepatocellular carcinoma
Source: BMC Cancer. 2010 Jul 6;10:356. doi: 10.1186/1471-2407-10-356 (PMC2915982; doi:10.1186/1471-2407-10-356)
Supplement: Additional file 2 — The result of Q-TOF mass spectrometry analysis of the band up in Figure 2. Peptide sequences identified from band up by Q-TOF analysis. [file 1471-2407-10-356-S2.PDF]

Mascot Search Results

```
User            : wang1
Email           : y_ying_77@yahoo.com.cn
Search title    :
MS data file    : D:\20020616lilei\WangQ-1.pkl
Database        : NCBInr 20050611 (2506589 sequences; 850049330 residues)
Taxonomy        : Homo sapiens (human) (134726 sequences)
Timestamp       : 16 Jun 2005 at 07:29:25 GMT
Significant hits: gi|16306978  Annexin A2, isoform 2 [Homo sapiens]
                  gi|31645     glyceraldehyde-3-phosphate dehydrogenase [Homo sapiens]
                  gi|500638    hnRNP protein A2 [Homo sapiens]
                  gi|28336     mutant beta-actin (beta'-actin) [Homo sapiens]
                  gi|36102     unnamed protein product [Homo sapiens]
                  gi|187281    M4 protein
```

Probability Based Mowse Score

Ions score is -10*Log(P), where P is the probability that the observed match is a random event.
Individual ions scores > 36 indicate identity or extensive homology (p<0.05).
Protein scores are derived from ions scores as a non-probabilistic basis for ranking protein hits.

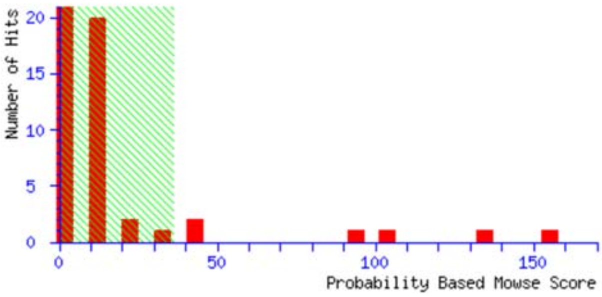

Peptide Summary Report

Format As: Peptide Summary   Help

Significance threshold p< 0.05   Max. number of hits AUTO

Standard scoring ◉ MudPIT scoring ○ Ions score cut-off 0 Show sub-sets ☐

Show pop-ups ◉ Suppress pop-ups ○ Sort unassigned Decreasing Score Require bold red ☐

Select All | Select None | Search Selected | ☐ **Error tolerant**

1. gi|16306978 **Mass:** 38594 **Score:** 155 **Queries matched:** 3
Annexin A2, isoform 2 [Homo sapiens]
☐ Check to include this hit in error tolerant search

	Query	Observed	Mr(expt)	Mr(calc)	Delta	Miss	Score	Expect	Rank	Peptide
☑	9	711.36	1420.71	1420.69	0.02	0	27	0.61	1	K.SLYYYIQQDTK.G
☑	10	730.85	1459.68	1459.67	0.02	0	56	0.00066	1	K.SYSPYDMLESIR.K
☑	11	771.94	1541.87	1541.84	0.02	0	72	8.4e-06	1	K.GVDEVTIVNILTNR.S

Proteins matching the same set of peptides:
gi|34364597 **Mass:** 40328 **Score:** 155 **Queries matched:** 3
hypothetical protein [Homo sapiens]
gi|50845388 **Mass:** 40386 **Score:** 155 **Queries matched:** 3
annexin A2 isoform 1 [Homo sapiens]
gi|56966699 **Mass:** 38638 **Score:** 155 **Queries matched:** 3
Chain A, Annexin A2: Does It Induce Membrane Aggregation By A New Multimeric State Of The Protein
gi|56967119 **Mass:** 36460 **Score:** 155 **Queries matched:** 3
Chain B, Structure Of Human Annexin A2 In The Presence Of Calcium Ions
gi|62202495 **Mass:** 38580 **Score:** 155 **Queries matched:** 3
ANXA2 protein [Homo sapiens]
gi|62896643 **Mass:** 38564 **Score:** 155 **Queries matched:** 3
annexin A2 isoform 2 variant [Homo sapiens]
gi|18645167 **Mass:** 38552 **Score:** 154 **Queries matched:** 3
Annexin A2, isoform 2 [Homo sapiens]

2. gi|31645 **Mass:** 36031 **Score:** 140 **Queries matched:** 2
glyceraldehyde-3-phosphate dehydrogenase [Homo sapiens]
☐ Check to include this hit in error tolerant search

	Query	Observed	Mr(expt)	Mr(calc)	Delta	Miss	Score	Expect	Rank	Peptide
☑	12	807.46	1612.91	1612.89	0.01	0	63	7e-05	1	K.LVINGNPITIFQER.D
☑	14	882.41	1762.81	1762.80	0.02	0	77	5.5e-06	1	K.LISWYDNEFGYSNR.V

Proteins matching the same set of peptides:
gi|35053 **Mass:** 35470 **Score:** 140 **Queries matched:** 2
uracil DNA glycosylase [Homo sapiens]
gi|32891805 **Mass:** 36030 **Score:** 140 **Queries matched:** 2
glyceraldehyde-3-phosphate dehydrogenase [Homo sapiens]

3. gi|500638 **Mass:** 35984 **Score:** 106 **Queries matched:** 3
hnRNP protein A2 [Homo sapiens]
☐ Check to include this hit in error tolerant search

	Query	Observed	Mr(expt)	Mr(calc)	Delta	Miss	Score	Expect	Rank	Peptide
☑	1	594.83	1187.65	1187.64	0.01	0	75	7.8e-06	1	K.IDTIEIITDR.Q
☑	4	689.33	1376.65	1376.62	0.02	0	31	0.17	1	R.GGGGNFGPGPGSNFR.G
	5	689.33	1376.65	1376.62	0.03	0	(2)	1.4e+02	5	R.GGGGNFGPGPGSNFR.G

Proteins matching the same set of peptides:
gi|62647260 **Mass:** 37407 **Score:** 106 **Queries matched:** 3
PREDICTED: similar to Heterogeneous nuclear ribonucleoproteins A2/B1 (hnRNP A2 / hnRNP B1) [Rattus norveg

4. gi|28336 **Mass:** 41786 **Score:** 93 **Queries matched:** 1
mutant beta-actin (beta'-actin) [Homo sapiens]
☐ Check to include this hit in error tolerant search

	Query	Observed	Mr(expt)	Mr(calc)	Delta	Miss	Score	Expect	Rank	Peptide
☑	15	895.96	1789.90	1789.88	0.02	0	93	1.2e-07	1	K.SYELPDGQVITIGNER.F

Proteins matching the same set of peptides:
gi|178027 **Mass:** 42081 **Score:** 93 **Queries matched:** 1
alpha-actin
gi|178045 **Mass:** 25862 **Score:** 93 **Queries matched:** 1
gamma-actin
gi|178067 **Mass:** 36783 **Score:** 93 **Queries matched:** 1
actin prepeptide
gi|998467 **Mass:** 3021 **Score:** 93 **Queries matched:** 1
48 kda histamine receptor subunit peptide 4 {internal fragment} [human, peripheral blood mononuclear cell
gi|6671507 **Mass:** 41982 **Score:** 93 **Queries matched:** 1
actin, alpha 2, smooth muscle, aorta [Mus musculus]
gi|12408252 **Mass:** 41989 **Score:** 93 **Queries matched:** 1
FKSG30 [Homo sapiens]
gi|12805167 **Mass:** 41850 **Score:** 93 **Queries matched:** 1
Actg2 protein [Mus musculus]
gi|14250401 **Mass:** 40978 **Score:** 93 **Queries matched:** 1
actin, beta [Homo sapiens]
gi|15277503 **Mass:** 40194 **Score:** 93 **Queries matched:** 1
ACTB protein [Homo sapiens]
gi|16306948 **Mass:** 17713 **Score:** 93 **Queries matched:** 1
Unknown (protein for IMAGE:3897065) [Homo sapiens]
gi|16359158 **Mass:** 41736 **Score:** 93 **Queries matched:** 1
Beta actin [Homo sapiens]
gi|16924319 **Mass:** 40477 **Score:** 93 **Queries matched:** 1
Unknown (protein for IMAGE:3538275) [Homo sapiens]
gi|17511847 **Mass:** 41766 **Score:** 93 **Queries matched:** 1
ACTG1 protein [Homo sapiens]
gi|21070355 **Mass:** 41710 **Score:** 93 **Queries matched:** 1
beta actin [Cavia porcellus]
gi|33563240 **Mass:** 42024 **Score:** 93 **Queries matched:** 1
actin, alpha 1, skeletal muscle [Mus musculus]

gi|40225338 **Mass:** 18439 **Score:** 93 **Queries matched:** 1
ACTG1 protein [Homo sapiens]
gi|40226101 **Mass:** 29393 **Score:** 93 **Queries matched:** 1
ACTG1 protein [Homo sapiens]
gi|49168516 **Mass:** 41898 **Score:** 93 **Queries matched:** 1
ACTG2 [Homo sapiens]
gi|56204817 **Mass:** 32028 **Score:** 93 **Queries matched:** 1
actin, alpha 1, skeletal muscle [Homo sapiens]
gi|56204818 **Mass:** 37800 **Score:** 93 **Queries matched:** 1
actin, alpha 1, skeletal muscle [Homo sapiens]
gi|62530430 **Mass:** 41992 **Score:** 93 **Queries matched:** 1
cardiac alpha actin 1 [Rana catesbeiana]
gi|62897409 **Mass:** 41696 **Score:** 93 **Queries matched:** 1
beta actin variant [Homo sapiens]
gi|62897625 **Mass:** 41738 **Score:** 93 **Queries matched:** 1
beta actin variant [Homo sapiens]
gi|62897671 **Mass:** 41694 **Score:** 93 **Queries matched:** 1
beta actin variant [Homo sapiens]
gi|63055057 **Mass:** 41976 **Score:** 93 **Queries matched:** 1
hypothetical protein LOC345651 [Homo sapiens]

---

5. gi|36102 **Mass:** 34131 **Score:** 44 **Queries matched:** 1
unnamed protein product [Homo sapiens]

☐ Check to include this hit in error tolerant search

	Query	Observed	Mr(expt)	Mr(calc)	Delta	Miss	Score	Expect	Rank	Peptide
☑	13	**850.40**	**1698.78**	**1698.75**	**0.03**	**0**	**44**	**0.0079**	**1**	**K.GFAFVTFDDHDSVDK.I**

Proteins matching the same set of peptides:
gi|37610 **Mass:** 20025 **Score:** 44 **Queries matched:** 1
unnamed protein product [Homo sapiens]
gi|133252 **Mass:** 22118 **Score:** 44 **Queries matched:** 1
Heterogeneous nuclear ribonucleoprotein A1 (Helix-destabilizing protein) (Single-strand binding protein) (
gi|133254 **Mass:** 38822 **Score:** 44 **Queries matched:** 1
Heterogeneous nuclear ribonucleoprotein A1 (Helix-destabilizing protein) (Single-strand binding protein) (
gi|2194069 **Mass:** 21002 **Score:** 44 **Queries matched:** 1
Hnrnp A1 (Rbd1,2) From Homo Sapiens
gi|2554653 **Mass:** 20784 **Score:** 44 **Queries matched:** 1
Up1, The Two Rna-Recognition Motif Domain Of Hnrnp A1
gi|13436308 **Mass:** 31733 **Score:** 44 **Queries matched:** 1
Unknown (protein for IMAGE:3615335) [Homo sapiens]
gi|14043070 **Mass:** 38723 **Score:** 44 **Queries matched:** 1
heterogeneous nuclear ribonucleoprotein A1 isoform b [Homo sapiens]
gi|20664272 **Mass:** 22249 **Score:** 44 **Queries matched:** 1
Chain A, Up1, The Two Rna-Recognition Motif Domain Of Hnrnp A1
gi|39654500 **Mass:** 20831 **Score:** 44 **Queries matched:** 1
Chain A, Crystal Structure Of Up1 Complexed With D(Tagg(6mi)ttaggg): A Human Telomeric Repeat Containing (
gi|41150277 **Mass:** 34279 **Score:** 44 **Queries matched:** 1
PREDICTED: similar to Heterogeneous nuclear ribonucleoprotein A1 (Helix-destabilizing protein) (Single-st
gi|41151081 **Mass:** 34236 **Score:** 44 **Queries matched:** 1
PREDICTED: similar to Heterogeneous nuclear ribonucleoprotein A1 (Helix-destabilizing protein) (Single-st
gi|47939618 **Mass:** 34159 **Score:** 44 **Queries matched:** 1
Heterogeneous nuclear ribonucleoprotein A1, isoform a [Homo sapiens]
gi|51467131 **Mass:** 27616 **Score:** 44 **Queries matched:** 1
PREDICTED: similar to Heterogeneous nuclear ribonucleoprotein A1 (Helix-destabilizing protein) (Single-st
gi|51474130 **Mass:** 25657 **Score:** 44 **Queries matched:** 1
PREDICTED: similar to Heterogeneous nuclear ribonucleoprotein A1 (Helix-destabilizing protein) (Single-st
gi|51492884 **Mass:** 33890 **Score:** 44 **Queries matched:** 1
PREDICTED: similar to Heterogeneous nuclear ribonucleoprotein A1 (Helix-destabilizing protein) (Single-st
gi|55957261 **Mass:** 34204 **Score:** 44 **Queries matched:** 1
OTTHUMP00000018460 [Homo sapiens]
gi|57106316 **Mass:** 34175 **Score:** 44 **Queries matched:** 1
PREDICTED: similar to Heterogeneous nuclear ribonucleoprotein A1 (Helix-destabilizing protein) (Single-st
gi|62897249 **Mass:** 30761 **Score:** 44 **Queries matched:** 1
heterogeneous nuclear ribonucleoprotein A1 isoform a variant [Homo sapiens]

---

6. gi|187281 **Mass:** 77555 **Score:** 44 **Queries matched:** 1
M4 protein

☐ Check to include this hit in error tolerant search

	Query	Observed	Mr(expt)	Mr(calc)	Delta	Miss	Score	Expect	Rank	Peptide
☑	3	**632.85**	**1263.68**	**1263.69**	**-0.00**	**0**	**45**	**0.0088**	**1**	**R.AFITNIPFDVK.W**

Proteins matching the same set of peptides:
gi|3126878 **Mass:** 73512 **Score:** 44 **Queries matched:** 1
M4 protein deletion mutant [Homo sapiens]
gi|16905456 **Mass:** 73572 **Score:** 44 **Queries matched:** 1
ribonucleoprotein [Homo sapiens]
gi|18044562 **Mass:** 77464 **Score:** 44 **Queries matched:** 1
Heterogeneous nuclear ribonucleoprotein M, isoform a [Homo sapiens]
gi|37747452 **Mass:** 73048 **Score:** 44 **Queries matched:** 1
Unknown (protein for MGC:64929) [Homo sapiens]

Peptide matches not assigned to protein hits: (no details means no match)

	Query	Observed	Mr(expt)	Mr(calc)	Delta	Miss	Score	Expect	Rank	Peptide
☑	2	**599.82**	**1197.63**	**1197.62**	**0.01**	**0**	**36**	**0.068**	**1**	**YPENFFLLR**
☑	7	**696.83**	**1391.65**	**1391.65**	**0.01**	**1**	**15**	**10**	**1**	**ATVEETARAADCR**
☑	5	689.33	1376.65	1376.72	-0.06	1	13	11	1	TFGQGTRVEINR
☑	6	**690.35**	**1378.68**	**1378.67**	**0.01**	**1**	**10**	**28**	**1**	**MVNKDMNGFPVK**
☑	17	**747.06**	**2238.15**	**2238.18**	**-0.02**	**1**	**10**	**19**	**1**	**IPRGQENQLVALIPYSDQR + Acetyl (N-term)**
☑	8	**471.23**	**1410.66**	**1410.66**	**-0.01**	**1**	**2**	**1.4e+02**	**1**	**KDGGETNYVDSVK**
☑	16	***742.39***	***2224.14***							
☑	18	***747.39***	***2239.15***							

Search Parameters

Type of search : MS/MS Ion Search
Enzyme : Trypsin
Variable modifications : Acetyl (N-term),Carbamidomethyl (C)
Mass values : Monoisotopic
Protein Mass : Unrestricted
Peptide Mass Tolerance : ± 50 ppm
Fragment Mass Tolerance: ± 0.1 Da
Max Missed Cleavages : 1
Instrument type : ESI-QUAD-TOF
Number of queries : 18

Mascot: http://www.matrixscience.com/
