# Supplementary material for: Up-regulation and subcellular localization of hnRNP A2/B1 in the development of hepatocellular carcinoma
Source: BMC Cancer. 2010 Jul 6;10:356. doi: 10.1186/1471-2407-10-356 (PMC2915982; doi:10.1186/1471-2407-10-356)
Supplement: Additional file 3 — The result of Q-TOF mass spectrometry analysis of the band down in Figure 2. Peptide sequences identified from band down by Q-TOF analysis. [file 1471-2407-10-356-S3.PDF]

Mascot Search Results

```
User            : wang2
Email           : y_ying_77@yahoo.com.cn
Search title    :
MS data file    : D:\20020616lilei\WangQ-2.pkl
Database        : NCBInr 20050611 (2506589 sequences; 850049330 residues)
Taxonomy        : Homo sapiens (human) (134726 sequences)
Timestamp       : 16 Jun 2005 at 07:30:33 GMT
Significant hits: gi|500638    hnRNP protein A2 [Homo sapiens]
                  gi|31092     unnamed protein product [Homo sapiens]
                  gi|460789    transformation upregulated nuclear protein [Homo sapiens]
                  gi|356168    histone H1b
                  gi|22770669  histone H1 [Homo sapiens]
                  gi|12804929  Mitochondrial malate dehydrogenase, precursor [Homo sapiens]
                  gi|28336     mutant beta-actin (beta'-actin) [Homo sapiens]
```

Probability Based Mowse Score

Ions score is -10*Log(P), where P is the probability that the observed match is a random event.
Individual ions scores > 36 indicate identity or extensive homology (p<0.05).
Protein scores are derived from ions scores as a non-probabilistic basis for ranking protein hits.

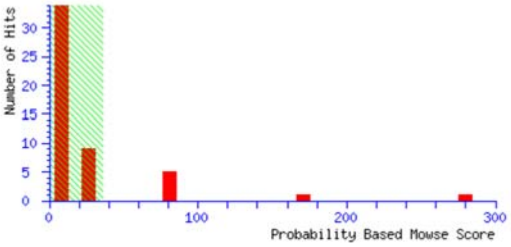

Peptide Summary Report

Format As: Peptide Summary — Help

Significance threshold p< 0.05 — Max. number of hits AUTO

Standard scoring (selected) — MudPIT scoring — Ions score cut-off 0 — Show sub-sets

Show pop-ups (selected) — Suppress pop-ups — Sort unassigned Decreasing Score — Require bold red

Select All — Select None — Search Selected — Error tolerant

1. gi|500638 **Mass:** 35984 **Score:** 279 **Queries matched:** 6
hnRNP protein A2 [Homo sapiens]
Check to include this hit in error tolerant search

Query	Observed	Mr(expt)	Mr(calc)	Delta	Miss	Score	Expect	Rank	Peptide
7	544.26	1086.50	1086.48	0.02	0	23	1.2	1	R.NYYEQWGK.L
9	594.83	1187.65	1187.64	0.01	0	76	7.5e-06	1	K.IDTIEIITDR.Q
13	689.33	1376.64	1376.62	0.01	0	48	0.0037	1	R.GGGGNFGPGPGSNFR.G
23	565.93	1694.77	1694.76	0.01	0	(12)	11	1	R.GFGFVTFDDHDPVDK.I
24	848.39	1694.77	1694.76	0.01	0	42	0.013	1	R.GFGFVTFDDHDPVDK.I
25	899.97	1797.92	1797.91	0.00	0	91	1.7e-07	1	K.LFIGGLSFETTEESLR.N

Proteins matching the same set of peptides:
gi|62647260 **Mass:** 37407 **Score:** 279 **Queries matched:** 6
PREDICTED: similar to Heterogeneous nuclear ribonucleoproteins A2/B1 (hnRNP A2 / hnRNP B1) [Rattus norvegicus]

---

2. gi|31092 **Mass:** 50095 **Score:** 166 **Queries matched:** 5
unnamed protein product [Homo sapiens]
Check to include this hit in error tolerant search

Query	Observed	Mr(expt)	Mr(calc)	Delta	Miss	Score	Expect	Rank	Peptide
3	488.28	974.55	974.54	0.00	0	41	0.019	1	R.LPLQDVYK.I
4	488.28	974.55	974.54	0.01	0	(24)	0.96	1	R.LPLQDVYK.I
5	513.31	1024.61	1024.60	0.01	0	56	0.00026	1	K.IGGIGTVPVGR.V
15	468.92	1403.72	1403.72	0.00	0	(46)	0.0074	1	K.YYVTIIDAPGHR.D
16	702.87	1403.73	1403.72	0.02	0	69	3e-05	1	K.YYVTIIDAPGHR.D

Proteins matching the same set of peptides:
gi|7108915 **Mass:** 46240 **Score:** 166 **Queries matched:** 5
glucocorticoid receptor AF-1 specific elongation factor [Homo sapiens]
gi|15277711 **Mass:** 42997 **Score:** 166 **Queries matched:** 5
translation elongation factor 1 alpha 1-like 14 [Homo sapiens]
gi|48734733 **Mass:** 50153 **Score:** 166 **Queries matched:** 5
Eukaryotic translation elongation factor 1 alpha 1 [Homo sapiens]
gi|48734966 **Mass:** 50091 **Score:** 166 **Queries matched:** 5

Eukaryotic translation elongation factor 1 alpha 1 [Homo sapiens]
gi|48735185 **Mass:** 47839 **Score:** 166 **Queries matched:** 5
EEF1A1 protein [Homo sapiens]
gi|55665593 **Mass:** 50153 **Score:** 166 **Queries matched:** 5
eukaryotic translation elongation factor 1 alpha-like 3 [Homo sapiens]
gi|57163863 **Mass:** 50109 **Score:** 166 **Queries matched:** 5
elongation factor 1 alpha [Felis catus]
gi|62896589 **Mass:** 50093 **Score:** 166 **Queries matched:** 5
eukaryotic translation elongation factor 1 alpha 1 variant [Homo sapiens]
gi|62896605 **Mass:** 50167 **Score:** 166 **Queries matched:** 5
eukaryotic translation elongation factor 1 alpha 1 variant [Homo sapiens]
gi|62896661 **Mass:** 50110 **Score:** 166 **Queries matched:** 5
eukaryotic translation elongation factor 1 alpha 1 variant [Homo sapiens]
gi|62897525 **Mass:** 50079 **Score:** 166 **Queries matched:** 5
eukaryotic translation elongation factor 1 alpha 1 variant [Homo sapiens]
gi|62897621 **Mass:** 50079 **Score:** 166 **Queries matched:** 5
eukaryotic translation elongation factor 1 alpha 1 variant [Homo sapiens]
gi|62897653 **Mass:** 50081 **Score:** 166 **Queries matched:** 5
eukaryotic translation elongation factor 1 alpha 1 variant [Homo sapiens]

---

3. gi|460789 **Mass:** 51040 **Score:** 87 **Queries matched:** 1
transformation upregulated nuclear protein [Homo sapiens]
☐ Check to include this hit in error tolerant search

	Query	Observed	Mr(expt)	Mr(calc)	Delta	Miss	Score	Expect	Rank	Peptide
☑	12	**670.91**	**1339.80**	**1339.80**	**0.01**	**0**	**87**	**1.5e-07**	**1**	**K.IILDLISESPIK.G**

Proteins matching the same set of peptides:
gi|16923998 **Mass:** 50944 **Score:** 87 **Queries matched:** 1
heterogeneous nuclear ribonucleoprotein K [Rattus norvegicus]
gi|38197650 **Mass:** 50996 **Score:** 87 **Queries matched:** 1
Hnrpk protein [Rattus norvegicus]
gi|51464712 **Mass:** 42939 **Score:** 87 **Queries matched:** 1
PREDICTED: similar to heterogeneous nuclear ribonucleoprotein K [Homo sapiens]
gi|55958543 **Mass:** 33955 **Score:** 87 **Queries matched:** 1
heterogeneous nuclear ribonucleoprotein K [Homo sapiens]
gi|55958544 **Mass:** 47528 **Score:** 87 **Queries matched:** 1
heterogeneous nuclear ribonucleoprotein K [Homo sapiens]
gi|55958547 **Mass:** 41781 **Score:** 87 **Queries matched:** 1
heterogeneous nuclear ribonucleoprotein K [Homo sapiens]
gi|59381084 **Mass:** 51026 **Score:** 87 **Queries matched:** 1
heterogeneous nuclear ribonucleoprotein K transcript variant [Homo sapiens]
gi|62088704 **Mass:** 48774 **Score:** 87 **Queries matched:** 1
heterogeneous nuclear ribonucleoprotein K isoform a variant [Homo sapiens]

---

4. gi|356168 **Mass:** 21721 **Score:** 82 **Queries matched:** 2
histone H1b
☐ Check to include this hit in error tolerant search

	Query	Observed	Mr(expt)	Mr(calc)	Delta	Miss	Score	Expect	Rank	Peptide
☑	10	**599.84**	**1197.67**	**1197.66**	**0.01**	**0**	**37**	**0.042**	**1**	**K.ASGPPVSELITK.A**
☑	21	**760.89**	**1519.76**	**1519.75**	**0.01**	**0**	**45**	**0.0078**	**1**	**-.SETAPAAPAAPAPAEK.T + Acetyl (N-term)**

Proteins matching the same set of peptides:
gi|9863668 **Mass:** 21852 **Score:** 82 **Queries matched:** 2
histone 1, H1e [Homo sapiens]
gi|66365795 **Mass:** 21880 **Score:** 82 **Queries matched:** 2
H1 histone family, member 4 [Homo sapiens]

---

5. gi|22770669 **Mass:** 21352 **Score:** 82 **Queries matched:** 2
histone H1 [Homo sapiens]
☐ Check to include this hit in error tolerant search

Query	Observed	Mr(expt)	Mr(calc)	Delta	Miss	Score	Expect	Rank	Peptide
10	599.84	1197.67	1197.66	0.01	0	37	0.042	1	K.ASGPPVSELITK.A
21	760.89	1519.76	1519.75	0.01	0	45	0.0078	1	M.SETAPAAPAAAPPAEK.A + Acetyl (N-term)

---

6. gi|12804929 **Mass:** 35509 **Score:** 78 **Queries matched:** 1
Mitochondrial malate dehydrogenase, precursor [Homo sapiens]
☐ Check to include this hit in error tolerant search

	Query	Observed	Mr(expt)	Mr(calc)	Delta	Miss	Score	Expect	Rank	Peptide
☑	22	**780.91**	**1559.81**	**1559.79**	**0.02**	**0**	**78**	**3.7e-06**	**1**	**K.VDFPQDQLTALTGR.I**

Proteins matching the same set of peptides:
gi|21735621 **Mass:** 35481 **Score:** 78 **Queries matched:** 1
mitochondrial malate dehydrogenase precursor [Homo sapiens]
gi|41472053 **Mass:** 33208 **Score:** 78 **Queries matched:** 1
unknown [Homo sapiens]

gi|49168580 **Mass:** 35537 **Score:** 78 **Queries matched:** 1
MDH2 [Homo sapiens]

---

7. gi|28336 **Mass:** 41786 **Score:** 77 **Queries matched:** 1
mutant beta-actin (beta'-actin) [Homo sapiens]

☐ Check to include this hit in error tolerant search

	Query	Observed	Mr(expt)	Mr(calc)	Delta	Miss	Score	Expect	Rank	Peptide
☑	20	758.86	1515.71	1515.70	0.02	0	77	4.6e-06	1	K.QEYDESGPSIVHR.K

Proteins matching the same set of peptides:

gi|178045 **Mass:** 25862 **Score:** 77 **Queries matched:** 1
gamma-actin
gi|825616 **Mass:** 13979 **Score:** 77 **Queries matched:** 1
unnamed protein product [Homo sapiens]
gi|12408252 **Mass:** 41989 **Score:** 77 **Queries matched:** 1
FKSG30 [Homo sapiens]
gi|14250401 **Mass:** 40978 **Score:** 77 **Queries matched:** 1
actin, beta [Homo sapiens]
gi|15277503 **Mass:** 40194 **Score:** 77 **Queries matched:** 1
ACTB protein [Homo sapiens]
gi|16306948 **Mass:** 17713 **Score:** 77 **Queries matched:** 1
Unknown (protein for IMAGE:3897065) [Homo sapiens]
gi|16359158 **Mass:** 41736 **Score:** 77 **Queries matched:** 1
Beta actin [Homo sapiens]
gi|16924319 **Mass:** 40477 **Score:** 77 **Queries matched:** 1
Unknown (protein for IMAGE:3538275) [Homo sapiens]
gi|17511847 **Mass:** 41766 **Score:** 77 **Queries matched:** 1
ACTG1 protein [Homo sapiens]
gi|21070355 **Mass:** 41710 **Score:** 77 **Queries matched:** 1
beta actin [Cavia porcellus]
gi|40225338 **Mass:** 18439 **Score:** 77 **Queries matched:** 1
ACTG1 protein [Homo sapiens]
gi|40226101 **Mass:** 29393 **Score:** 77 **Queries matched:** 1
ACTG1 protein [Homo sapiens]
gi|51460457 **Mass:** 117666 **Score:** 77 **Queries matched:** 1
PREDICTED: similar to POTE2A [Homo sapiens]
gi|51460787 **Mass:** 67263 **Score:** 77 **Queries matched:** 1
PREDICTED: similar to FKSG30 [Homo sapiens]
gi|51493213 **Mass:** 41995 **Score:** 77 **Queries matched:** 1
PREDICTED: similar to FKSG30 [Homo sapiens]
gi|62897409 **Mass:** 41696 **Score:** 77 **Queries matched:** 1
beta actin variant [Homo sapiens]
gi|62897625 **Mass:** 41738 **Score:** 77 **Queries matched:** 1
beta actin variant [Homo sapiens]
gi|62897671 **Mass:** 41694 **Score:** 77 **Queries matched:** 1
beta actin variant [Homo sapiens]

Peptide matches not assigned to protein hits: (no details means no match)

	Query	Observed	Mr(expt)	Mr(calc)	Delta	Miss	Score	Expect	Rank	Peptide
☑	11	630.82	1259.63	1259.62	0.01	0	32	0.2	1	YLFLGDYVDR
☑	30	1042.01	2082.01	2081.94	0.07	1	28	0.31	1	NLDHGKNVNEGDGFEYFK
☑	1	421.76	841.51	841.50	0.01	0	27	0.4	1	GITLSVRP
☑	14	699.88	1397.75	1397.78	-0.03	1	23	1.2	1	NDLKLIEGVDIK + Acetyl (N-term)
☑	6	541.82	1081.63	1081.59	0.04	0	11	8.4	1	VAGLGNDPLAR
☑	28	971.47	1940.93	1940.94	-0.01	1	10	19	1	CVCNSCGLEIVDKYLLK + Acetyl (N-term)
☑	2	435.78	869.54	869.51	0.03	1	10	11	1	IRSLSPR + Acetyl (N-term)
☑	18	728.39	1454.77	1454.71	0.06	0	10	24	1	STSGGTYALGCLVK + Acetyl (N-term); Carbamidomethyl (C)
☑	17	717.84	1433.67	1433.69	-0.02	0	9	29	1	VEGNFNPFASPQK
☑	34	766.06	2295.17	2295.16	0.01	1	7	38	1	AGGFISFNGSWRVQGILAMSR + Acetyl (N-term)
☑	19	746.35	1490.69	1490.72	-0.03	1	4	87	1	APQACCTLDKTVGK + Carbamidomethyl (C)
☑	32	1106.06	2210.12	2210.06	0.05	1	4	75	1	TVNLTDFSNKWDSTNPFPK
☑	8	564.84	1127.66	1127.64	0.02	1	4	74	1	LGVSVSPSRAR
☑	29	999.49	1996.97	1997.03	-0.05	0	2	1.2e+02	1	ISVGLPVGAVINCADNTGAK + Acetyl (N-term); Carbamidomethyl (C)
☑	33	747.06	2238.15	2238.11	0.04	1	2	1.1e+02	1	IFEPNCLDAFPNLKDFISR
☑	31	737.71	2210.11	2210.08	0.04	0	2	1.3e+02	1	EGGLPEGPVLEALLCAETGEK + Acetyl (N-term); Carbamidomethyl (C)
☑	35	855.40	2563.19	2563.32	-0.13	0	0	1.5e+02	1	LNLHLAIFDPEVLAAMNGPEVMK + Acetyl (N-term)
☑	26	*970.97*	*1939.93*							
☑	27	*970.97*	*1939.93*							
☑	36	*877.09*	*2628.25*							
☑	37	*898.42*	*2692.24*							
☑	38	*955.45*	*2863.34*							
☑	39	*717.09*	*2864.32*							
☑	40	*991.18*	*2970.53*							
☑	41	*1135.57*	*3403.69*							

Search Parameters

```
Type of search         : MS/MS Ion Search
Enzyme                 : Trypsin
Variable modifications : Acetyl (N-term),Carbamidomethyl (C)
Mass values            : Monoisotopic
Protein Mass           : Unrestricted
Peptide Mass Tolerance : ± 50 ppm
Fragment Mass Tolerance: ± 0.1 Da
Max Missed Cleavages   : 1
Instrument type        : ESI-QUAD-TOF
Number of queries      : 41
```

Mascot: http://www.matrixscience.com/
