# Supplementary material for: Up-regulation and subcellular localization of hnRNP A2/B1 in the development of hepatocellular carcinoma
Source: BMC Cancer. 2010 Jul 6;10:356. doi: 10.1186/1471-2407-10-356 (PMC2915982; doi:10.1186/1471-2407-10-356)
Supplement: Additional file 4 — The result of Q-TOF mass spectrometry analysis of the spot up in Figure 3. Peptide sequences identified from spot up by Q-TOF analysis. [file 1471-2407-10-356-S4.PDF]

Analysis Information

Report Type	Protein-Peptide Summary by Spot
Sample Set Name	lilei-cuihq
Analysis Name	New Analysis 4
Reported By	08/17/2005 15:31:33 - admin

Analysis Type	Combined (MS+MS/MS)
Database	
Creation Date	08/17/2005 15:13:17
Last Modified	08/17/2005 15:29:01
MS Acq. : Proc. Methods	(Unspecified) : (Unspecified)
Interpretation Method	(Unspecified)

		**Instr./Spot Origin**	AB347000090/lilei20050812	**Process Status**	Analysis Succeeded
**Plate [#] Name**	[1] 000300003908_5	**4700 Sample Name**		**Spectra**	9

Rank	Protein Name	Accession No.	Protein Score	Protein C. I. %	Total Ion Score	Total Ion C. I. %	Species	Protein MW	Protein PI	Pep. Count	MS Ion Intensity	Intensity Matched	Best Ion Score	Best Ion C. I. %
1	hnRNP protein A2 [Homo sapiens]	gi|500638	163	100	57	99.996		35983.9	8.67	14	1472126.5	19.436	35	99.427

Peptide Information

Calc. Mass	Obsrv. Mass	± da	± ppm	Start Seq.	End Seq.	Sequence	Ion Score	C. I. %	Modification
993.4855	993.4878	0.0023	2	35	42	LTDCVVMR			1 Carbamidomethyl (C)
1013.4434	1013.45	0.0066	7	192	201	GGNFGFGDSR			
1013.4434	1013.45	0.0066	7	192	201	GGNFGFGDSR	22	88.972	
1050.4414	1050.469	0.0276	26	118	125	DYFEEYGK			
1165.5232	1165.5428	0.0196	17	109	117	EDTEEHHLR			
1188.647	1188.6555	0.0085	7	126	135	IDTIEIITDR			
1221.5527	1221.5723	0.0196	16	179	188	QEMQEVQSSR			
1338.7012	1338.7227	0.0215	16	88	100	EESGKPGAHVTVK			
1377.6293	1377.6433	0.014	10	202	216	GGGGNFGPGPGSNFR			
1377.6293	1377.6433	0.014	10	202	216	GGGGNFGPGPGSNFR	35	99.427	
1410.6873	1410.7568	0.0695	49	162	173	YHTINGHNAEVR			
1695.7649	1695.7837	0.0188	11	142	156	GFGFVTFDDHDPVDK			
1798.9221	1798.9421	0.02	11	11	26	LFIGGLSFETTEESLR			
1927.017	1927.0397	0.0227	12	10	26	KLFIGGLSFETTEESLR			
2189.9053	2189.9343	0.029	13	314	338	NMGGPYGGGNYGPGGS GGSGGYGGR			
2189.9053	2189.9343	0.029	13	314	338	NMGGPYGGGNYGPGGS GGSGGYGGR			

2205.9001	2205.915	0.0149	7	314	338	NMGGPYGGGNYGPGGS GGSGGYGGR			1 Oxidation (M)
2277.155	2277.0662	-0.0888	-39	142	161	GFGFVTFDDHDPVDKIVL QK			

2	PREDICTED: similar to Heterogeneous nuclear ribonucleoproteins A2/B1 (hnRNP A2 / hnRNP B1) [Rattus	gi|62647260	161	100	57	99.996		37406.7	8.97	14	1472126.5	19.436	35	99.427

Peptide Information

Calc. Mass	Obsrv. Mass	± da	± ppm	Start Seq.	End Seq.	Sequence	Ion Score	C. I. %	Modification
993.4855	993.4878	0.0023	2	47	54	LTDCVVMR			1 Carbamidomethyl (C)
1013.4434	1013.45	0.0066	7	204	213	GGNFGFGDSR			
1013.4434	1013.45	0.0066	7	204	213	GGNFGFGDSR	22	88.972	
1050.4414	1050.469	0.0276	26	130	137	DYFEEYGK			
1165.5232	1165.5428	0.0196	17	121	129	EDTEEHHLR			
1188.647	1188.6555	0.0085	7	138	147	IDTIEIITDR			
1221.5527	1221.5723	0.0196	16	191	200	QEMQEVQSSR			
1338.7012	1338.7227	0.0215	16	100	112	EESGKPGAHVTVK			
1377.6293	1377.6433	0.014	10	214	228	GGGGNFGPGPGSNFR			
1377.6293	1377.6433	0.014	10	214	228	GGGGNFGPGPGSNFR	35	99.427	
1410.6873	1410.7568	0.0695	49	174	185	YHTINGHNAEVR			
1695.7649	1695.7837	0.0188	11	154	168	GFGFVTFDDHDPVDK			
1798.9221	1798.9421	0.02	11	23	38	LFIGGLSFETTEESLR			
1927.017	1927.0397	0.0227	12	22	38	KLFIGGLSFETTEESLR			
2189.9053	2189.9343	0.029	13	326	350	NMGGPYGGGNYGPGGS GGSGGYGGR			
2189.9053	2189.9343	0.029	13	326	350	NMGGPYGGGNYGPGGS GGSGGYGGR			
2205.9001	2205.915	0.0149	7	326	350	NMGGPYGGGNYGPGGS GGSGGYGGR			1 Oxidation (M)
2277.155	2277.0662	-0.0888	-39	154	173	GFGFVTFDDHDPVDKIVL QK			

3	glyceraldehyde-3-phosphate dehydrogenase [Homo sapiens]	gi|31645	94	100	34	99.361		36031.4	8.26	9	1472126.5	23.244	34	99.361

Peptide Information

Calc. Mass	Obsrv. Mass	± da	± ppm	Start Seq.	End Seq.	Sequence	Ion Score	C. I. %	Modification
1411.7903	1411.7305	-0.0598	-42	201	215	GALQNIIPASTGAAK			
1411.7903	1411.7305	-0.0598	-42	201	215	GALQNIIPASTGAAK			
1530.7944	1530.8071	0.0127	8	235	248	VPTANVSVVDLTCR	34	99.361	1 Carbamidomethyl (C)
1530.7944	1530.8071	0.0127	8	235	248	VPTANVSVVDLTCR			1 Oxidation (M)
1613.9009	1613.9172	0.0163	10	67	80	LVINGNPITIFQER			
1763.8022	1763.8247	0.0225	13	310	323	LISWYDNEFGYSNR			
1833.9197	1833.9402	0.0205	11	146	162	IISNASCTTNCLAPLAK			2 Carbamidomethyl (C)
2213.1091	2213.1316	0.0225	10	119	139	VIISAPSADAPMFVMGVN HEK			
2229.1042	2229.1284	0.0242	11	119	139	VIISAPSADAPMFVMGVN HEK			1 Oxidation (M)
2277.0378	2277.0662	0.0284	12	87	107	WGDAGAEYVVESTGVFT			

						[illegible]
2369.2104	2369.2312	0.0208	9	118	139	RVIISAPSADAPMFVMGV NHEK
2595.3599	2595.3882	0.0283	11	163	186	VIHDNFGIVEGLMTTVHAI TATQK
