# Supplementary material for: Up-regulation and subcellular localization of hnRNP A2/B1 in the development of hepatocellular carcinoma
Source: BMC Cancer. 2010 Jul 6;10:356. doi: 10.1186/1471-2407-10-356 (PMC2915982; doi:10.1186/1471-2407-10-356)
Supplement: Additional file 5 — The result of Q-TOF mass spectrometry analysis of the spot down in Figure 3. Peptide sequences identified from spot down by Q-TOF analysis. [file 1471-2407-10-356-S5.PDF]

Plate [#] Name	[1] 000300003908_5	Instr./Spot Origin	AB347000090/lilei20050812	Process Status	Analysis Succeeded
		4700 Sample Name		Spectra	9

Rank	Protein Name	Accession No.	Protein Score	Protein C. I. %	Total Ion Score	Total Ion C. I. %	Species	Protein MW	Protein PI	Pep. Count	MS Ion Intensity	Intensity Matched	Best Ion Score	Best Ion C. I. %
1	hnRNP protein A2 [Homo sapiens]	gi|500638	474	100	309	100		35983.9	8.67	18	2763186.75	69.614	77	100

Peptide Information

Calc. Mass	Obsrv. Mass	± da	± ppm	Start Seq.	End Seq.	Sequence	Ion Score	C. I. %	Modification
993.4855	993.4767	-0.0088	-9	35	42	LTDCVVMR			1 Carbamidomethyl (C)
1009.4805	1009.4606	-0.0199	-20	35	42	LTDCVVMR			1 Carbamidomethyl (C),1 Oxidation (M)
1013.4434	1013.4346	-0.0088	-9	192	201	GGNFGFGDSR			
1013.4434	1013.4346	-0.0088	-9	192	201	GGNFGFGDSR	77	100	
1050.4414	1050.4244	-0.017	-16	118	125	DYFEEYGK	40	99.854	
1050.4414	1050.4244	-0.017	-16	118	125	DYFEEYGK			

1087.4843	1087.4757	-0.0086	-8	27	34	NYYEQWGK			
1165.5232	1165.5142	-0.009	-8	109	117	EDTEEHHLR			
1188.647	1188.6422	-0.0048	-4	126	135	IDTIEIITDR			
1188.647	1188.6422	-0.0048	-4	126	135	IDTIEIITDR	32	99.076	
1221.5527	1221.5474	-0.0053	-4	179	188	QEMQEVQSSR			
1237.5477	1237.5354	-0.0123	-10	179	188	QEMQEVQSSR			1 Oxidation (M)
1338.7012	1338.7112	0.01	7	88	100	EESGKPGAHVTVK			
1377.6293	1377.6349	0.0056	4	202	216	GGGGNFGPGPGSNFR			
1377.6293	1377.6349	0.0056	4	202	216	GGGGNFGPGPGSNFR	70	100	
1410.6873	1410.6885	0.0012	1	162	173	YHTINGHNAEVR			
1434.7079	1434.7184	0.0105	7	35	47	LTDCVVMRDPASK			
1695.7649	1695.7623	-0.0026	-2	142	156	GFGFVTFDDHDPVDK	21	87.598	
1695.7649	1695.7623	-0.0026	-2	142	156	GFGFVTFDDHDPVDK			2 Carbamidomethyl (C)
1798.9221	1798.9265	0.0044	2	11	26	LFIGGLSFETTEESLR			
1798.9221	1798.9265	0.0044	2	11	26	LFIGGLSFETTEESLR	28	97.658	
1851.866	1851.8658	-0.0002	0	141	156	RGFGFVTFDDHDPVDK			
1879.9659	1879.9739	0.008	4	102	117	LFVGGIKEDTEEHHLR			
1927.017	1927.0199	0.0029	2	10	26	KLFIGGLSFETTEESLR			
2189.9053	2189.9104	0.0051	2	314	338	NMGGPYGGGNYGPGGS GGSGGYGGR	41	99.891	
2189.9053	2189.9104	0.0051	2	314	338	NMGGPYGGGNYGPGGS GGSGGYGGR			1 Oxidation (M)
2205.9001	2205.906	0.0059	3	314	338	NMGGPYGGGNYGPGGS GGSGGYGGR			2 Carbamidomethyl (C)
2495.0393	2495.0449	0.0056	2	227	254	GFGDGYNGYGGGPGGG NFGGSPGYGGGR			

2 PREDICTED: similar to Heterogeneous nuclear ribonucleoproteins A2/B1 (hnRNP A2 / hnRNP B1) [Rattus gi|62647260 471 100 309 100 37406.7 8.97 18 2763186.75 69.614 77 100

Peptide Information

Calc. Mass	Obsrv. Mass	± da	± ppm	Start Seq.	End Seq.	Sequence	Ion Score	C. I. %	Modification
993.4855	993.4767	-0.0088	-9	47	54	LTDCVVMR			1 Carbamidomethyl (C)
1009.4805	1009.4606	-0.0199	-20	47	54	LTDCVVMR			1 Carbamidomethyl (C),1 Oxidation (M)
1013.4434	1013.4346	-0.0088	-9	204	213	GGNFGFGDSR			
1013.4434	1013.4346	-0.0088	-9	204	213	GGNFGFGDSR	77	100	
1050.4414	1050.4244	-0.017	-16	130	137	DYFEEYGK	40	99.854	
1050.4414	1050.4244	-0.017	-16	130	137	DYFEEYGK			1 Oxidation (M)
1087.4843	1087.4757	-0.0086	-8	39	46	NYYEQWGK			
1165.5232	1165.5142	-0.009	-8	121	129	EDTEEHHLR			
1188.647	1188.6422	-0.0048	-4	138	147	IDTIEIITDR			
1188.647	1188.6422	-0.0048	-4	138	147	IDTIEIITDR	32	99.076	
1221.5527	1221.5474	-0.0053	-4	191	200	QEMQEVQSSR			
1237.5477	1237.5354	-0.0123	-10	191	200	QEMQEVQSSR			1 Oxidation (M)
1338.7012	1338.7112	0.01	7	100	112	EESGKPGAHVTVK			
1377.6293	1377.6349	0.0056	4	214	228	GGGGNFGPGPGSNFR			
1377.6293	1377.6349	0.0056	4	214	228	GGGGNFGPGPGSNFR	70	100	

1410.6873	1410.6885	0.0012	1	174	185	YHTINGHNAEVR			
1434.7079	1434.7184	0.0105	7	47	59	LTDCVVMRDPASK			
1695.7649	1695.7623	-0.0026	-2	154	168	GFGFVTFDDHDPVDK	21	87.598	
1695.7649	1695.7623	-0.0026	-2	154	168	GFGFVTFDDHDPVDK			
1798.9221	1798.9265	0.0044	2	23	38	LFIGGLSFETTEESLR			
1798.9221	1798.9265	0.0044	2	23	38	LFIGGLSFETTEESLR	28	97.658	
1851.866	1851.8658	-0.0002	0	153	168	RGFGFVTFDDHDPVDK			
1879.9659	1879.9739	0.008	4	114	129	LFVGGIKEDTEEHHLR			
1927.017	1927.0199	0.0029	2	22	38	KLFIGGLSFETTEESLR			
2189.9053	2189.9104	0.0051	2	326	350	NMGGPYGGGNYGPGGS GGSGGYGGR	41	99.891	
2189.9053	2189.9104	0.0051	2	326	350	NMGGPYGGGNYGPGGS GGSGGYGGR			2 Carbamidomethyl (C),2 Oxidation (M)
2205.9001	2205.906	0.0059	3	326	350	NMGGPYGGGNYGPGGS GGSGGYGGR			2 Carbamidomethyl (C),2 Oxidation (M)
2495.0393	2495.0449	0.0056	2	239	266	GFGDGYNGYGGGPGGG NFGGSPGYGGGR			

3	HNRPA2B1 protein [Homo sapiens]	gi|31455238	231	100	121	100		28394.1	4.79	13	2763186.75	27.5	40	99.854

Peptide Information

Calc. Mass	Obsrv. Mass	± da	± ppm	Start Seq.	End Seq.	Sequence	Ion Score	C. I. %	Modification
993.4855	993.4767	-0.0088	-9	35	42	LTDCVVMR			1 Carbamidomethyl (C)
1009.4805	1009.4606	-0.0199	-20	35	42	LTDCVVMR			1 Carbamidomethyl (C),1 Oxidation (M)
1050.4414	1050.4244	-0.017	-16	118	125	DYFEEYGK	40	99.854	
1050.4414	1050.4244	-0.017	-16	118	125	DYFEEYGK			2 Carbamidomethyl (C)
1087.4843	1087.4757	-0.0086	-8	27	34	NYYEQWGK			
1165.5232	1165.5142	-0.009	-8	109	117	EDTEEHHLR			
1188.647	1188.6422	-0.0048	-4	126	135	IDTIEIITDR			
1188.647	1188.6422	-0.0048	-4	126	135	IDTIEIITDR	32	99.076	
1338.7012	1338.7112	0.01	7	88	100	EESGKPGAHVTVK			
1410.6873	1410.6885	0.0012	1	162	173	YHTINGHNAEVR			
1434.7079	1434.7184	0.0105	7	35	47	LTDCVVMRDPASK			
1695.7649	1695.7623	-0.0026	-2	142	156	GFGFVTFDDHDPVDK	21	87.598	
1695.7649	1695.7623	-0.0026	-2	142	156	GFGFVTFDDHDPVDK			1 Oxidation (M)
1798.9221	1798.9265	0.0044	2	11	26	LFIGGLSFETTEESLR			
1798.9221	1798.9265	0.0044	2	11	26	LFIGGLSFETTEESLR	28	97.658	
1851.866	1851.8658	-0.0002	0	141	156	RGFGFVTFDDHDPVDK			
1879.9659	1879.9739	0.008	4	102	117	LFVGGIKEDTEEHHLR			
1927.017	1927.0199	0.0029	2	10	26	KLFIGGLSFETTEESLR			
