# Supplementary material for: Up-regulation and subcellular localization of hnRNP A2/B1 in the development of hepatocellular carcinoma
Source: BMC Cancer. 2010 Jul 6;10:356. doi: 10.1186/1471-2407-10-356 (PMC2915982; doi:10.1186/1471-2407-10-356)
Supplement: Additional file 6 — The statistical analysis results of Table 1and Table 2. The SAS statistical analysis results. [file 1471-2407-10-356-S6.PDF]

The FREQ Procedure

Table of a by b

a / b<br>Frequency<br>Percent<br>Row Pct<br>Col Pct	1	2	Total
1	6<br>37.50<br>100.00<br>100.00	0<br>0.00<br>0.00<br>0.00	6<br>37.50
2	0<br>0.00<br>0.00<br>0.00	10<br>62.50<br>100.00<br>100.00	10<br>62.50
Total	6<br>37.50	10<br>62.50	16<br>100.00

Statistics for Table of a by b

Statistic	DF	Value	Prob
Chi-Square	1	16.0000	<.0001
Likelihood Ratio Chi-Square	1	21.1700	<.0001
Continuity Adj. Chi-Square	1	12.0178	0.0005
Mantel-Haenszel Chi-Square	1	15.0000	0.0001
Phi Coefficient		1.0000	
Contingency Coefficient		0.7071	
Cramer's V		1.0000	

Fisher's Exact Test

Cell (1,1) Frequency (F)	6
Left-sided Pr <= F	1.0000
Right-sided Pr >= F	1.249E-04
Table Probability (P)	1.249E-04
Two-sided Pr <= P	1.249E-04

Sample Size = 16

The FREQ Procedure

Table of a by b

a / b<br>Frequency<br>Percent<br>Row Pct<br>Col Pct	1	2	Total
1	6<br>10.00<br>54.55<br>100.00	5<br>8.33<br>45.45<br>9.26	11<br>18.33
2	0<br>0.00<br>0.00<br>0.00	49<br>81.67<br>100.00<br>90.74	49<br>81.67
Total	6<br>10.00	54<br>90.00	60<br>100.00

Statistics for Table of a by b

Statistic	DF	Value	Prob
Chi-Square	1	29.6970	<.0001
Likelihood Ratio Chi-Square	1	23.8518	<.0001
Continuity Adj. Chi-Square	1	23.9456	<.0001
Mantel-Haenszel Chi-Square	1	29.2020	<.0001
Phi Coefficient		0.7035	
Contingency Coefficient		0.5754	
Cramer's V		0.7035	

Fisher's Exact Test

Cell (1,1) Frequency (F)	6
Left-sided Pr <= F	1.0000
Right-sided Pr >= F	9.228E-06
Table Probability (P)	9.228E-06
Two-sided Pr <= P	9.228E-06

Sample Size = 60

The NPAR1WAY Procedure

Wilcoxon Scores (Rank Sums) for Variable b
Classified by Variable a

a	N	Sum of Scores	Expected Under H0	Std Dev Under H0	Mean Score
1	22	412.50	660.0	61.004235	18.7500
2	17	510.00	510.0	57.134297	30.0000
3	20	847.50	600.0	59.716601	42.3750

Average scores were used for ties.

Kruskal-Wallis Test

Chi-Square	21.6770
DF	2
Pr > Chi-Square	<.0001
